# Evaluating rice for salinity using pot-culture provides a systematic tolerance assessment at the seedling stage

**DOI:** 10.1186/s12284-019-0317-7

**Published:** 2019-07-30

**Authors:** Naqeebullah Kakar, Salah H. Jumaa, Edilberto Diaz Redoña, Marilyn L. Warburton, K. Raja Reddy

**Affiliations:** 1 Department of Plant and Soil Sciences, Mississippi State University, Mississippi State, MS 39762 USA; 2 Delta Research and Extension Center, Mississippi State University, 82 Stoneville Road, Stoneville, MS 38776 USA; 3 Corn Host Plant Resistance Research Unit, Crop Science Research Laboratory, USDA-ARS, Mississippi State, MS 39762 USA

**Keywords:** Morpho-physiological traits, Pot-culture screening, Principal component analysis (PCA), Rice (*Oryza sativa* L.), Salt stress response indices (SSRI), Salinity tolerance, Seedling stage

## Abstract

**Background:**

Rice (*Oryza sativa* L*.)* is one of the major staple food crops consumed globally. However, rice production is severely affected by high salinity levels, particularly at the seedling stage. A good solution would be the development of an efficient screening methodology to identify genotypes possessing genes for salt tolerance.

**Result:**

A new salinity tolerance screening technique using rice seedlings in pot-culture was tested. This method controls soil heterogeneity by using pure sand as a growth medium and minimizes unexpected extreme weather conditions with a movable shelter. Seventy-four rice genotypes were screened at three salinity treatments including high salt stress (electrical conductivity (EC) 12 dSm^− 1^), moderate salt stress (EC 6 dSm^− 1^), and control (no salt stress), imposed 1 week after emergence. Several shoot and root morpho-physiological traits were measured at 37 days after sowing. A wide range of variability was observed among genotypes for measured traits with root traits being identified as the best descriptors for tolerance to salt stress conditions. Salt stress response indices (SSRI) were used to classify the 74 rice genotypes; 7 genotypes (9.46%) were identified as salt sensitive, 27 (36.48%) each as low and moderately salt tolerant, and 13 (17.57%) as highly salt tolerant. Genotypes FED 473 and IR85427 were identified as the most salt tolerant and salt sensitive, respectively. These results were further confirmed by principal component analysis (PCA) for accuracy and reliability.

**Conclusion:**

Although tolerant genotypes still need to be confirmed in field studies and tolerance mechanisms identified at the molecular level, information gained from this study could help rice breeders and other scientists to accelerate breeding by selecting appropriate donor parents, progenies and potential genotypes at early growth stages necessary for salinity tolerance research.

**Electronic supplementary material:**

The online version of this article (10.1186/s12284-019-0317-7) contains supplementary material, which is available to authorized users.

## Background

Rice (*Oryza sativa* L*.)* is one of the major staple crops, consumed by more than half of the world’s population (Dawe et al. 2010). Production of rice must be increased quantitatively and improved qualitatively to meet the requirements of the growing population in the twenty-first century and to maintain global food security. Although rice has a wide geographic distribution extending from 50 N to 35S, it is vulnerable to climatic changes leading to low rice productivity. The rapidly changing climate is causing different abiotic stresses, including periods of drought, frequent floods, sea water inundations, etc. (Jagadish et al. 2012), which reduce the yield potential of current rice varieties. Among abiotic threats, salinity is the second most devastating constraint in rice production after drought, affecting approximately 1 billion ha of land globally (Fageria et al. 2012). This equals more than 6% of the world’s total farming area (Ismail and Horie 2017) and nearly 20% of the globally irrigated area (Munns 2002). Salinity in arable land is mainly caused by the excessive use of irrigation water with improper drainage, poor quality irrigation water containing an excess level of salts, and flooding from seawater (Ismail et al. 2007).

Salinity is also increasing in the United States, particularly in Louisiana, which is the third largest rice producing state in the country (USDA 2013). Because of its proximity to the Gulf of Mexico, water intrusion can easily occur in coastal areas during the hurricane seasons, making the land more vulnerable to increasing salinity. In California, salinity is also increasing, mainly due to irrigation practices at the seedling growth stage under the direct water-seeded system, the dominant irrigation system for rice production (Scardaci et al. 1996).

Rice is most sensitive to salt stress at the seedling and early vegetative stages (Lutts et al. 1995), and later at the reproductive stages (Ismail et al. 2007; Singh et al. 2009). Excess salt in soil adversely affects plant growth, development, and productivity when osmotic stress reduces water uptake by roots, (Munns and Tester 2008). Direct accumulation of salts disturbs metabolic processes and all major morpho-physiological and yield-related traits including tiller number, panicle length, spikelet number per panicle (Khatun et al. 1995), grain filling (Rao et al. 2013), plant biomass (Zeng et al. 2007) and photosynthesis (Ismail et al. 2007; Baker 2008), leading to significantly decreased yield.

Natural variation is an integral resource for the improvement of beneficial traits which can be found in both wild and domesticated germplasm. Exploiting existing natural variation can lead to an improvement in salt tolerance while maintaining good levels of agronomically and economically important traits like quality and yield. The study of natural variation can also improve understanding of the physiology and genetic mechanisms behind tolerance at sensitive stages of growth (Ismail and Horie 2017). Similarly, extensive and reliable phenotypic evaluation of cultivars is crucial to determine the extent of the genetic basis of salinity tolerance, or for dissecting component traits associated with tolerance, and subsequent exploitation via breeding (Ismail and Horie 2017).

Tolerance to salt stress depends on multiple morphological and physiological component traits. Previous studies have shown that among the physiological parameters, chlorophyll content, alterations in chlorophyll fluorescence (Fv/Fm), and membrane permeability are efficient potential indicators to determine the inhibitory effect of salinity on photosynthetic efficiency (Baker 2008). Similarly, different morphological parameters including leaf area, tiller number, panicle length, root length, dry weight, biomass, relative growth rate, and relative water content have been used to evaluate rice cultivars at the morphological and physiological (morpho-physiological) level for salinity tolerance (Zeng and Shannon 1998). Thus, assessing the cumulative effect of morpho-physiological traits can help to build a comprehensive protocol to evaluate rice genotypes and understand plant mechanisms for salinity tolerance.

Salinity tolerance in rice has been a target for improvement by rice breeders over the years. Evaluating genotypes to be advanced during breeding at an early growth stage via high-throughput phenotyping saves time and resources compared to traditional phenotyping strategies (Ismail and Horie 2017). Two key factors in identifying salt tolerant genotypes are an assessment of salinity sensitive growth stages (Lutts et al. 1995) and growth parameters associated with salinity tolerance (Ashraf and McNeilly 1987). Past morphological variables used in screening and described as effective salinity indices include shoot length, root length, plant biomass, and shoot Na^+^/K^+^ ratio (Zeng et al. 2007; Gregorio and Senadhira 1993).

Approaches to screening rice for salt tolerance include on-field mass screening and controlled environment screening using hydroponics or other artificial media (Ismail and Horie 2017). On-field mass screening without replication or multiple years and environments is not reliable for identification of suitable cultivars because of varying environmental factors (weather conditions, soil heterogeneity, and amount of salt accumulation in the soil; Ismail and Horie 2017). Most of the work on salinity tolerance, particularly at the seedling stage, has been done in the laboratory or greenhouses under controlled conditions using solutions of NaCl or mixtures of NaCl + CaCl (Flowers and Yeo 1988). Greenhouse screening with solution culture was initially thought to be advantageous over field screening because of controlled environmental conditions. However, solution culture does not truly represent field conditions and genotypes identified as salt tolerant in solution may not be so in the field, where the level of salinity may show a larger level of spatial and temporal variation (Tavakkoli 2011; Kopittke et al. 2011).

Thus, a proper understanding of the quantitative impacts and critical response thresholds for newly developed cultivars is still limited, particularly under conditions more representative of the field. Development of an intermediate, efficient, reliable, reproducible, and simple high throughput screening technique will improve the practical screening of salinity tolerance, particularly at early growth stages. Pot-culture screening under natural environmental conditions at early growth stages is a simple and rapid screening method and has been used to screen germplasm for salinity tolerance in other crops (Shannon 1997), but further confirmation is lacking.

In the present study, we designed a new screening technique using pot-culture where we controlled soil heterogeneity using pure sand as the growth medium and minimized unexpected extreme weather conditions by using a movable canopy when needed, yet simulated field conditions on other days by removing the canopy. We hypothesized that the selected rice genotypes would show a wide range of variability in morpho-physiological parameters in actual response to salt stress, making this screening methodology the first important step in screening and selection of better rice varieties, paving the way to exploitation of desirable genotypic variation in rice breeding programs for salinity tolerance. The objectives of the study were to (a) determine the quantitative effects of different levels of salinity stress on the selected rice genotypes; (b) identify the most important morpho-physiological descriptors of salinity and their critical threshold responses at an early growth stage; and (c) explore the genetic potential of 74 rice genotypes for salt tolerance and cluster them into different salinity groups, based on root and shoot morpho-physiological parameters.

## Results

Data for shoot and root morphological and physiological parameters for all rice genotypes (Additional file 1: Table S1) used in the current study were analyzed. Analysis of variance (*P* ≤ 0.001) revealed both significant and non-significant differences among the rice genotypes, salinity treatments, and their interaction (genotypes x salt stress) for all measured morpho-physiological shoot and root parameters (Table 1). This significance can be exploited for breeding, and genotype x salinity level interactions need to be considered when studying varieties under salt-stressed conditions.

**Table 1:** Analysis of variance across the 74 tropical rice genotype, treatments and their interaction for the morpho-physiological traits measured at the final harvest, 37 days after sowing; plant height (PH), tillers number (TN), leaf area (LA), longest root length (LRL), total root length (TRL), root surface area (SA), average diameter (AD), root volume (RV), root tips (TP), forks (FR), crossings (CR), chlorophyll (CH), flavonoids (FLV), anthocyanin (ANT), and nitrogen balance index (NBI)

Source	PH	TN	LA	LRL	TRL	SA	AD	RV	TP	FR	CR	CH	FLV	ANT	NBI
Salinity (S)	***	***	***	***	***	***	***	***	***	***	***	***	NS	***	***
Genotypes (G)	***	***	***	***	***	***	***	***	***	***	***	***	***	**	***
S * G	***	***	***	***	***	***	***	***	*	*	NS	NS	NS	NS	NS

Significant level ***, **,*, and N. S means *P*-value ˂ 0.001, 0.01, 0.05, and not significant, respectively

### Shoot growth and developmental parameters

Shoot growth and developmental parameters including plant height (PH), tiller numbers (TN) and leaf area (LA) were significantly different among rice genotypes, salinity treatments, and salinity x genotype interaction (Additional file 1: Table S2). PH was significantly higher at optimum (control) salinity level compared to moderate (6 dSm^− 1^) and high salinity levels (12 dSm^− 1^), ranging from 30.25 cm (IR86052) to 13.0 cm (IR70213). At moderate salinity stress, PH ranged from 22.71 cm (IR86052) to 8.33 cm (IRRI 123), with an average of 14.43 cm. However, at high salinity stress, PH was reduced significantly for all rice genotypes, ranging from 19.33 cm (IR86052) to 5.83 cm (IR05N412), with an average PH of 14.43 cm (Additional file 1: Table S2).

Tiller number (TN) and leaf area (LA) also followed the same trend and were significantly reduced at the high salinity level compared to the optimum salinity level. Average TN at optimum salinity level was 12, with a maximum of 20 and a minimum of 4 tillers in genotypes IR07F287 and IR10A134, respectively. Average TN was reduced to 6 tillers per plant at moderate salinity levels, with maximum and minimum ranging from 11 (IR86635) to 2 (75–1-127), respectively. At high salinity levels, TN was reduced significantly to an average of 4 tillers per plant, with a maximum of 7 (WAB) and a minimum of 1 (75–1-127) tillers per plant, respectively. Leaf area (LA) was reduced drastically, particularly under high salinity levels for all rice genotypes, and ranged from 560.65 cm^2^ (CT18614) to 13.5 cm^2^ (75–1-127), with an average of 174.5 cm^2^ compared to LA at optimum salinity levels, which varied from 1427.1 cm^2^ (IR86635) to 185.8 cm^2^ (IR10A134) with an average of 721.1 cm^2^ per plant. At moderate salinity levels, maximum (879.6 cm^2^ plant^− 1^) and minimum (105.4 cm^2^ plant^− 1^) LA was observed in genotypes IR86635 and IR09L179, respectively (Additional file 1: Table S2).

**Fig. 1:**
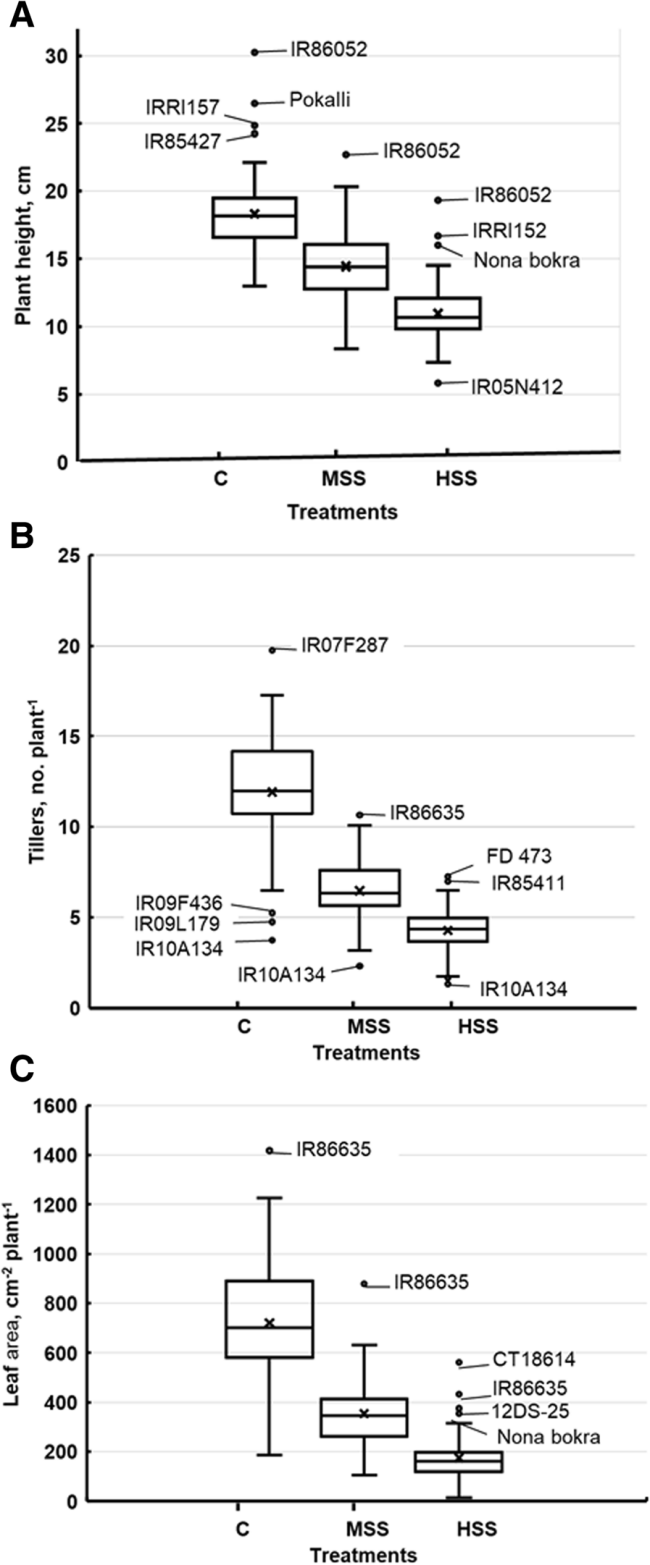
Box and whisker plots for shoot growth and developmental traits showing natural variation and the effect of different salinity treatments on the average (**a**) plant height, cm (**b**) tillers, no. plant^− 1^, and (**c**) leaf area, cm^2^ plant^− 1^ The whisker below the box represents the first quartile (Q1) or the fifth percentile showing the first 25% of data distribution in this range whereas the whisker above the box represents the third quartile (Q3) or 95th percentile showing the last 25% of the data distribution. The length of the box is called interquartile range (IQR) or (25th to 75th percentile), shows 25 to 75% of the data distribution for that particular trait and the horizontal line in the box indicates the median value. The genotypes below the first quartile or above the third quartile are representing outliers in individual traits, are also indicated

Substantial natural variation was observed for measured shoot growth, and developmental parameters at different salinity conditions and genotypes responded differently to the salinity treatments, showing great variability among the genotypes. On average, salinity treatments negatively affected recorded shoot growth and developmental traits (Fig. 1).

### Root growth and developmental parameters

Major root growth parameters including total root length (TRL), longest root length (LRL), root surface area (SA), average root diameter (AD) and root volume (RV) were significantly different among rice genotypes, salinity treatments, and salinity x genotype interaction (Additional file 1: Table S3). The average TRL decreased significantly from 5968.3 cm at optimum salinity levels to 4817.8 cm at high salinity levels; at moderate salinity levels average TRL was 5286.3 cm. TRL ranged from 7577.5 (Geumg) to 3201.3 cm (IR10A134) at optimum; 6698.4 (IR07F102) to 2818.8 cm (IR1A134) at moderate; and 7516.7 (BR47) to 669.3 cm (75–1-127) at high salinity levels.

Similarly, the LRL was also significantly reduced from 43.1 cm under optimum conditions to 41.2 and 33.0 cm at moderate and high salinity conditions, respectively. Root surface area decreased with the increase of salinity for all genotypes. Hence, the average SA at optimum conditions decreased from 1246.7 cm^− 2^ to 1025.6 and 679.0 cm^− 2^ when salinity was increased to 6 dSm^− 1^ and 12 dSm^− 1^, respectively. The decreasing trend was also observed in AD and RV parameters with increasing salinity concentrations. The mean optimum AD (0.7 mm) decreased to 0.6 mm at moderate and 0.4 mm at high salinity levels. RV was profoundly affected by high salt stress, and mean RV decreased from 21.9 cm^− 3^ under optimum conditions to 8.2 cm^− 3^ at high salinity levels (electrical conductivity 12 dSm^− 1^). Under high salt stress, RV ranged from 21.3 cm^− 3^ in genotype CT18245 to 0.8 cm^− 3^ in genotype 75–1-127, which is significantly less than under optimum conditions, which ranged from 37.6 mm (IRRI 157) to 5.5 mm (IR10A134) (Additional file 1: Table S3).

Major root developmental parameters, including root tips (TP), root forks (FR), and root crossings (CR) were significantly affected by salinity in most of the rice genotypes (Additional file 1: Table S4). The average number of TP under optimum conditions was 33308, with maximum (41336) and minimum (20850) TP expressed in genotypes IR86635 and IR10A134, respectively. However, at high salt stress, average TP decreased significantly to 30282.8 and genotypes BR47 and 75–1-127 expressed the highest (45566) and lowest (5476) number of TP, respectively. Similarly, the number of FR and CR were also significantly affected as the level of salinity increased from optimum to high (12 dSm^− 1^), decreasing the overall mean of FR and CR from 108884.8 and 7759.8 under control conditions to 72119.2 and 6239.3 at high salinity level, respectively.

**Fig. 2:**
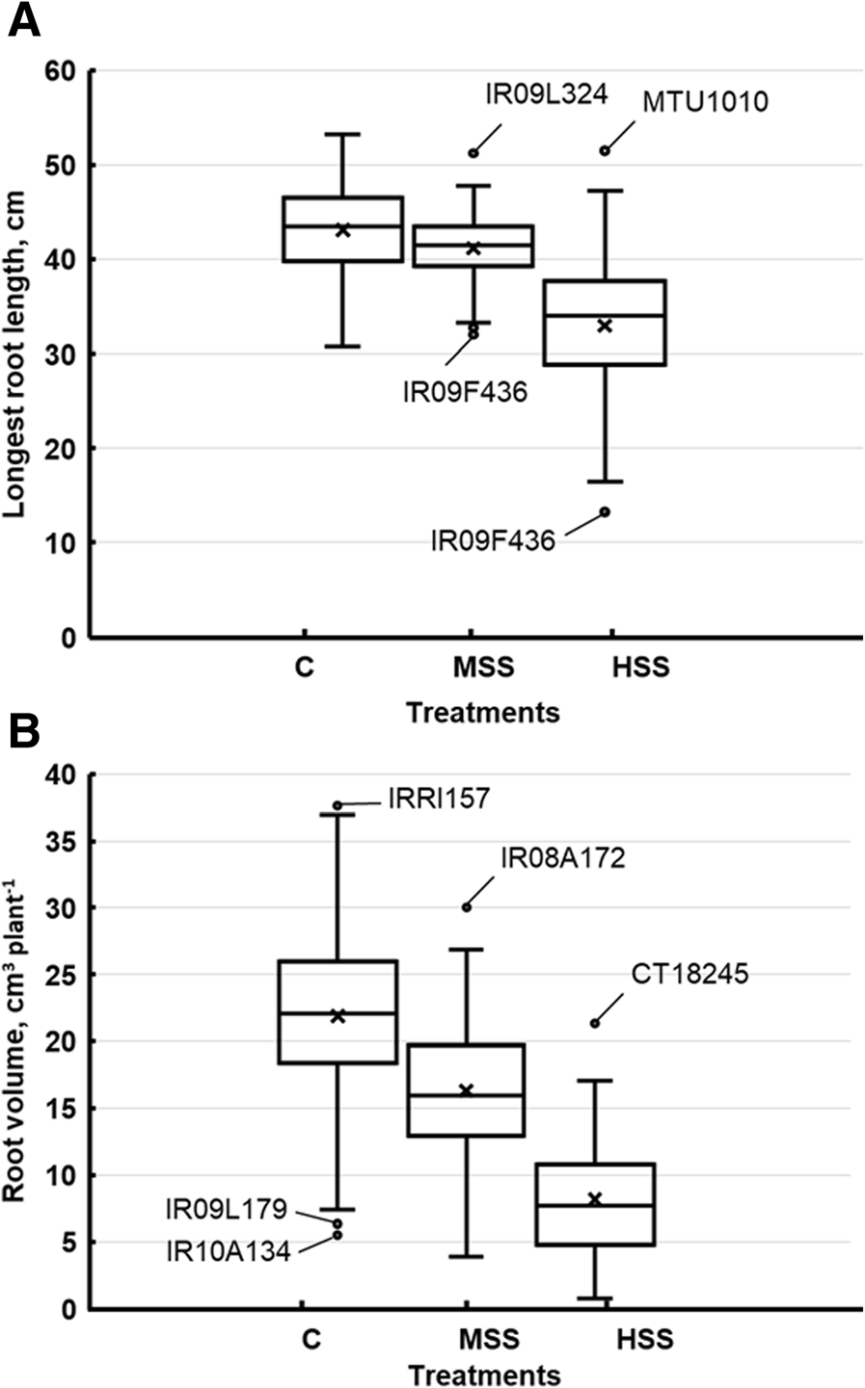
Box and whisker plots for root growth and development traits showing natural variation and the effect of different salinity treatments on the average (**a**) longest root length, cm plant^− 1^ and (**b**) root volume, cm^3^ plant^− 1^. The genotypes below the first quartile or above the third quartile are representing outliers in individual traits, are also indicated

Overall, higher numbers of TR, FR and CR were observed in known salt tolerant genotypes (BR47 and Geumg), indicating that salt tolerant genotypes develop extensive root systems, whereas the known salt sensitive genotype (75–1-127) showed the least TR, FR, and CR indicating a less vigorous root system under salinity stress (Additional file 1: Table S4). Wide natural variation was observed among the measured root growth and developmental traits at different salinity levels, and genotypes responded differently to the salinity treatments showing the presence of substantial genetic variability among the genotypes. On average, salinity treatments negatively affected the recorded root growth and developmental traits (Fig. 2).

### Physiological parameters

Among the major physiological parameters presented in Additional file 1: Table S5, flavonoids (FLV) and anthocyanins (ANT) showed non-significant differences among the genotypes at high salinity levels; however, chlorophyll content (CH) and ANT were significantly different (*P* > 0.001) between control and moderate salinity levels. Unlike other physiological parameters, nitrogen balance index (NBI) was found to be significantly different among rice genotypes at all three treatment conditions. Interestingly, genotype X salinity interaction was non-significant for all physiological parameters under optimum and both salt treatments. Mean CH contents increased from 21.4 μg cm^− 2^ at the optimum condition to 24.9 μg cm^− 2^ at moderate salinity levels but declined to 19.7 μg cm^− 2^ at high salinity levels. A similar trend was also observed in the average NBI with an increase from 20.2 to 22.7 at moderate salinity levels and decrease to 18.3 at high salinity levels. However, no significant changes were observed in average FL and ANT between the two salinity levels (Additional file 1: Table S5).

**Fig. 3:**
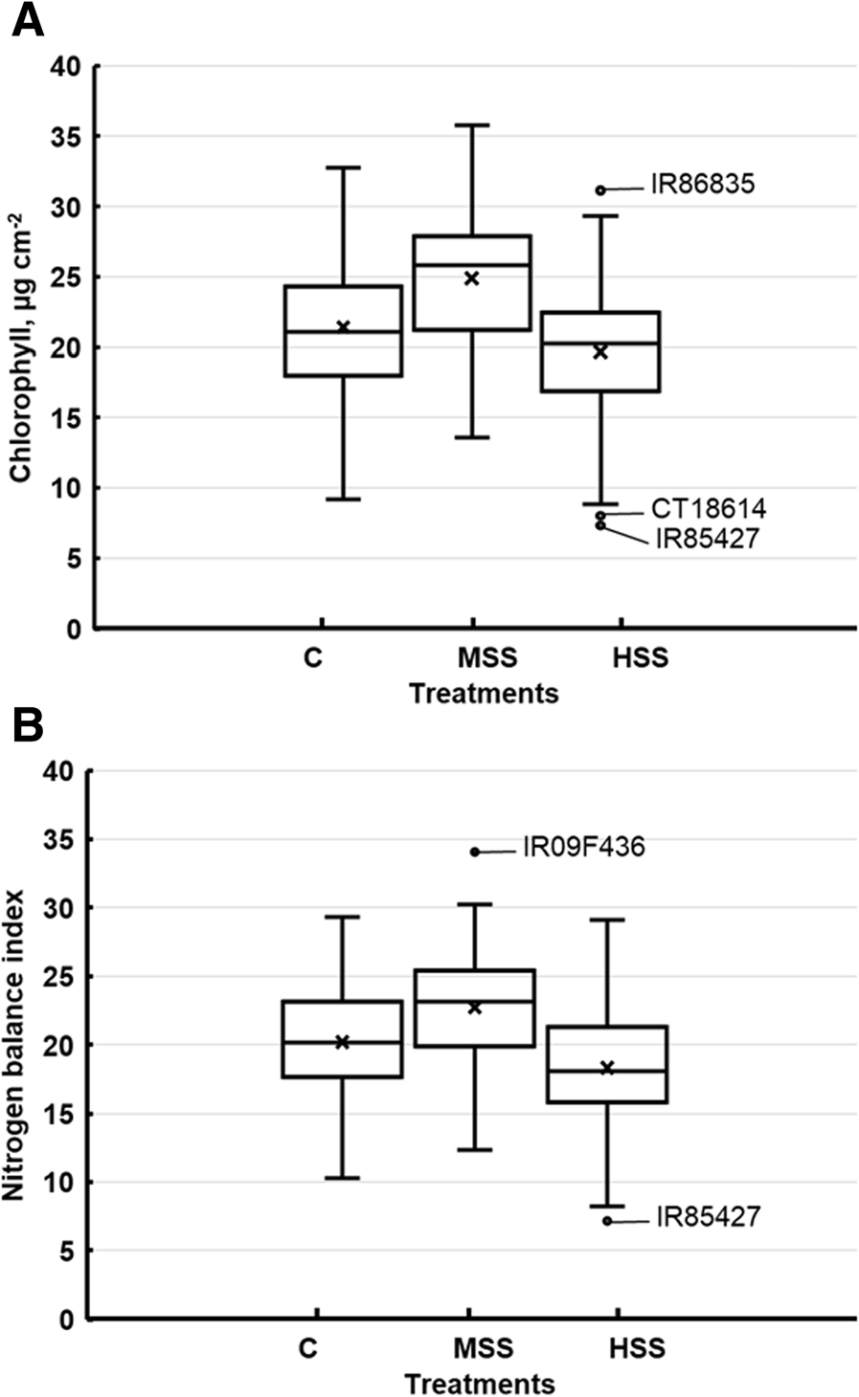
Box and whisker plots for physiological traits showing natural variation and the effect of different salinity treatments on the average (**a**) chlorophyll content, μg cm^− 2^, and (**b**) nitrogen balance index (unitless). The genotypes below the first quartile or above the third quartile are representing outliers in individual traits, are also indicated

Substantial natural variation was also observed among the measured physiological parameters at different salinity levels, and genotypes responded differently to the salinity treatments showing the presence of substantial genetic variability among the genotypes. On average, salinity treatments positively affected physiological traits at medium salinity levels but significantly reduced them at high salt stress (Fig. 3).

### Classification of rice genotypes based on SSRI and root image acquisition

**Fig. 4:**
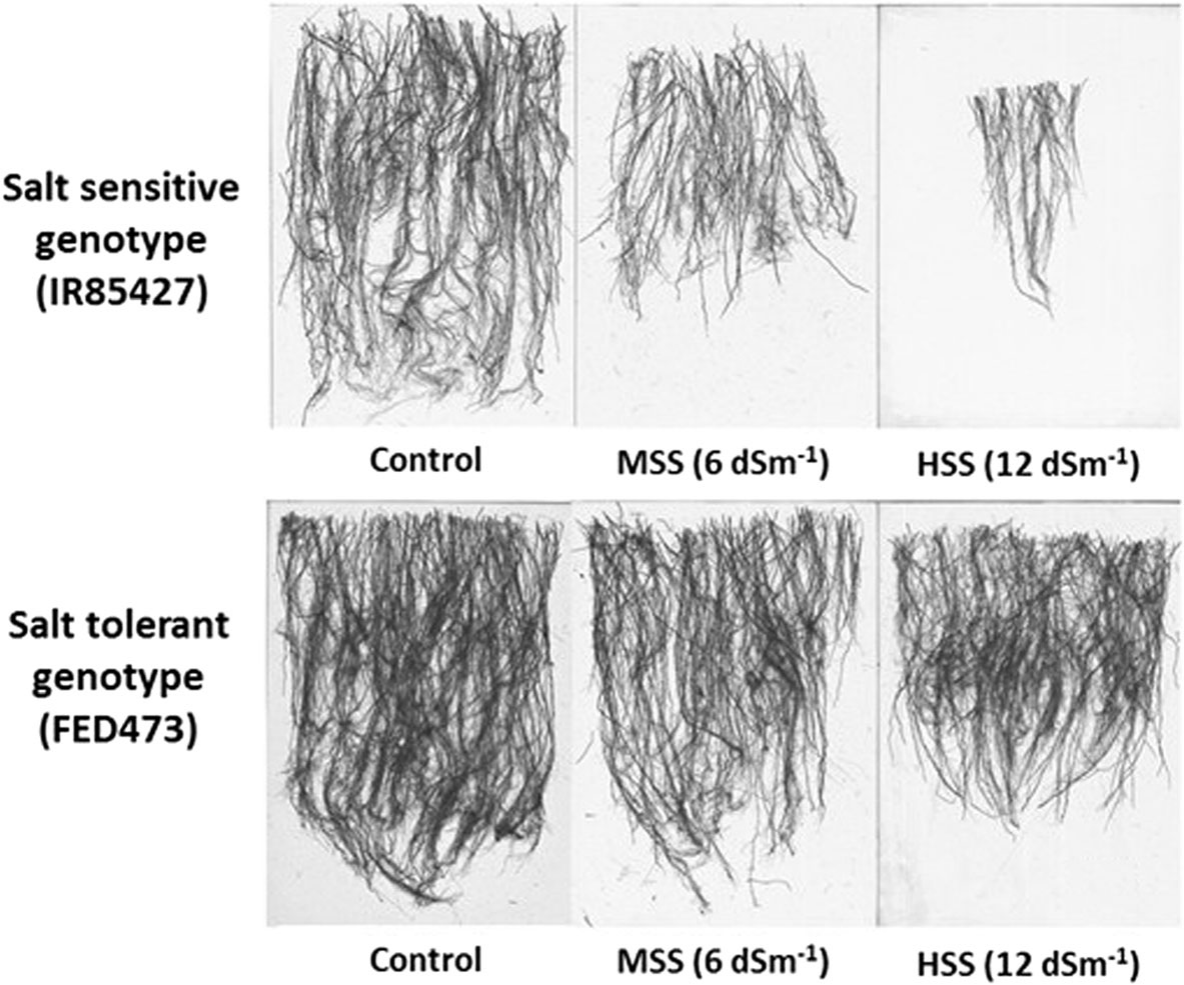
Representative scanned root images of salt-sensitive (**a**) and salt tolerant (**b**) rice genotypes, at three different salinity levels [control, moderate salt stress (MSS), and high salt stress (HSS), respectively

The total salt stress response index (TSSRI) values of all measured shoot, root, and physiological parameters at an early growth stage and their standard deviations were used to classify rice genotypes into four response groups (Table 2). Of the 74 rice genotypes, seven (9.46%) were identified as salt sensitive, and 26 (35%) had low, 27 (36%) had moderate, and 13 (17.57%) had high salt tolerance. TSSRI values for salt tolerance varied from 25.15 for genotype IR85427, identified as highly salt sensitive (Fig. 4a) to 39.87 for genotype FED 473, identified as highly salt tolerant (Fig. 4b).

**Table 2:** Classification of 74 rice genotypes using total salt stress response indices (TSSRI) of morpho-physiological parameters at the seedling stage. Values of TSSRI are given in the parenthesis

Salt Sensitive	Low Salt Tolerant	Moderate Salt Tolerant	High Salt Tolerant
(25.15–26.54)	(26.55–29.32)	(29.33–32.10)	(32.11–34.88)
IR85427 (25.15)	IR65482 (26.77)	IRRI 157 (29.40)	Pokalli (32.21)
75–1-127 (25.34)	IR74371 (27.68)	IR6-PAK (29.40)	IR86174 (32.39)
IR86126 (25.42)	IR06N155 (27.78)	IR09A130 (29.49)	MIL 240 (32.75)
IRRI 152 (25.68)	IR78222 (27.82)	MTU1010 (29.55)	HHZ 1 (33.11)
IR09F436 (26.04)	IR07F287 (27.89)	Geumg (30.01)	IR10A134 (33.62)
CT18372 (26.29)	IR86052 (27.97)	IR86174 (30.05)	PALMAR (34.01)
CT18237 (26.30)	CT18614 (28.07)	Rex (30.14)	IR85411 (34.27)
IR78221 (26.75)	IRRI 123 (28.10)	IR49830 (30.15)	IR08N136 (34.47)
	IR78049 (28.11)	CT18615 (30.21)	CT18245 (34.48)
	IR08A172 (28.11)	CT18244 (30.26)	N. B (35.14)
	IR09N537 (28.14)	IR85422 (30.28)	CT18233 (36.39)
	IR04A115 (28.41)	IR70213 (30.48)	IRRI 154 (36.88)
	CT6946 (28.49)	IR05F102 (30.60)	FED 473 (39.87)
	IR09L179 (28.60)	IR86635 (30.60)	
	HHZ 12 (28.68)	IR09L337 (30.79)	
	IR05N412 (28.73)	IR93323 (30.95)	
	IR75483 (28.79)	IR07K142 (31.02)	
	IR09L324 (28.90)	BR47 (31.09)	
	FED CARE (28.93)	12DS-25 (31.14)	
	IR10N230 (28.94)	IR64-NIL (31.16)	
	Apo (28.99)	IR86174 (31.17)	
	COL XXI (29.07)	IR88633 (31.19)	
	Thad (29.09)	CT18247 (31.23)	
	12DS-15 (29.26)	WAB (31.53)	
	IR65600 (29.31)	IR07F102 (31.89)	
	CT19561 (29.31)	FEDE 21 (31.95)	
		FED 2000 (32.16)	
8 (11%)	26 (35%)	27 (36%)	13 (18%)

**Fig. 5:**
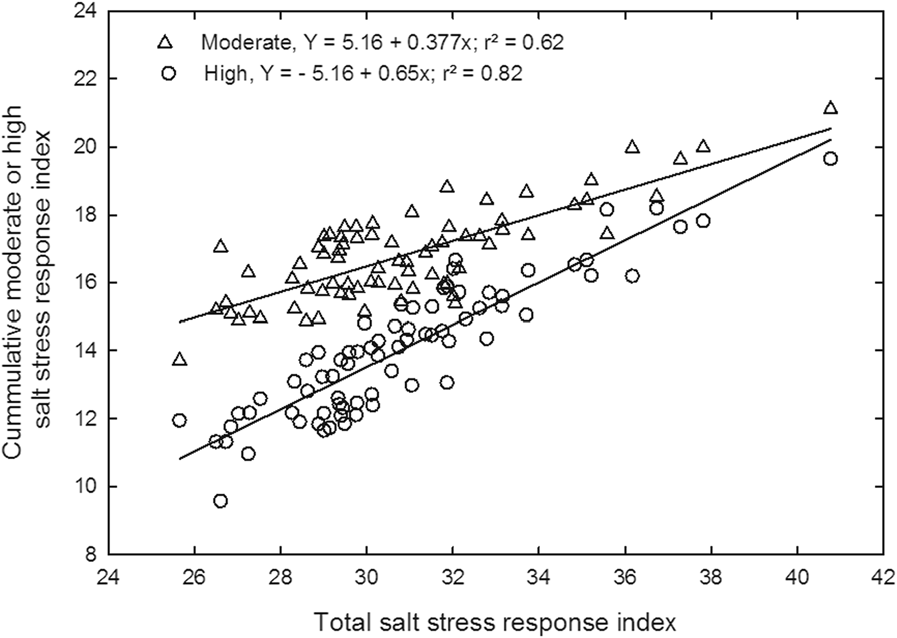
Relationship of total salt stress response index with the total shoot, root, and physiological salt stress response index for all the rice genotypes. Measurements were taken 37 days after sowing

**Fig. 6:**
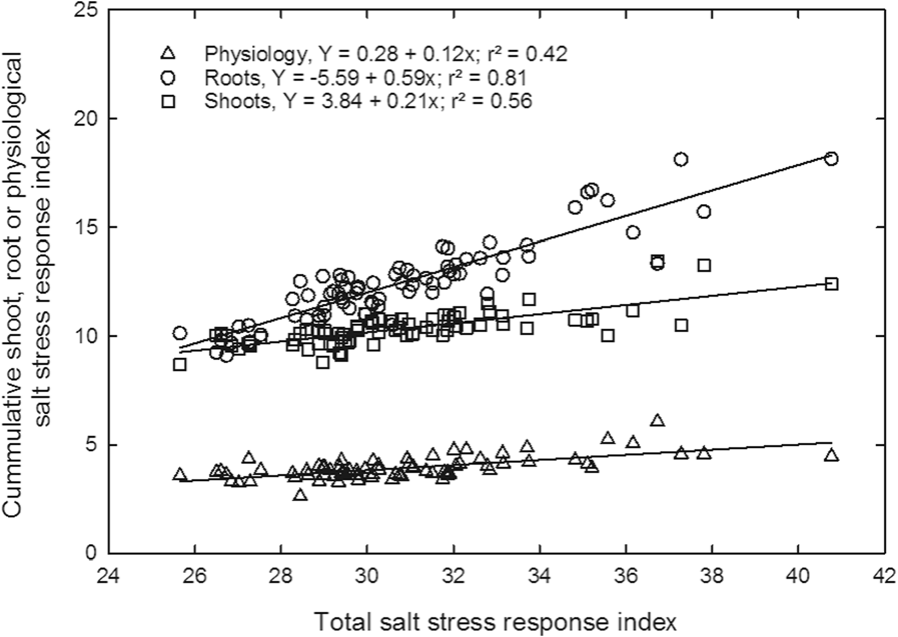
Relationship of total salt stress response index with cumulative moderate and cumulative high salt stress response indices for all the rice genotypes. Measurements were taken 37 days after sowing

TSSRI were also used to calculate the correlation between shoot, root, and physiological parameters for salt tolerance. The value of the coefficient of determination (R^2^) gives the percentage of variation of tolerance index explained by each independent variable. An overall positive correlation was observed between total salt stress response index and total shoot (R^2^ = 0.42), root (R^2^ = 0.81) and physiological parameters (R^2^ = 0.56) (Fig. 5). Similarly, TSSRI was also observed to be positively correlated with cumulative moderate (R^2^ = 0.62) and cumulative high salt stress response (R^2^ = 0.82) indices (at *P* = 0.0001, *n* = 74) (Fig. 6).

### Assessment of salt tolerant genotypes using principal component analysis (PCA)

**Fig. 7:**
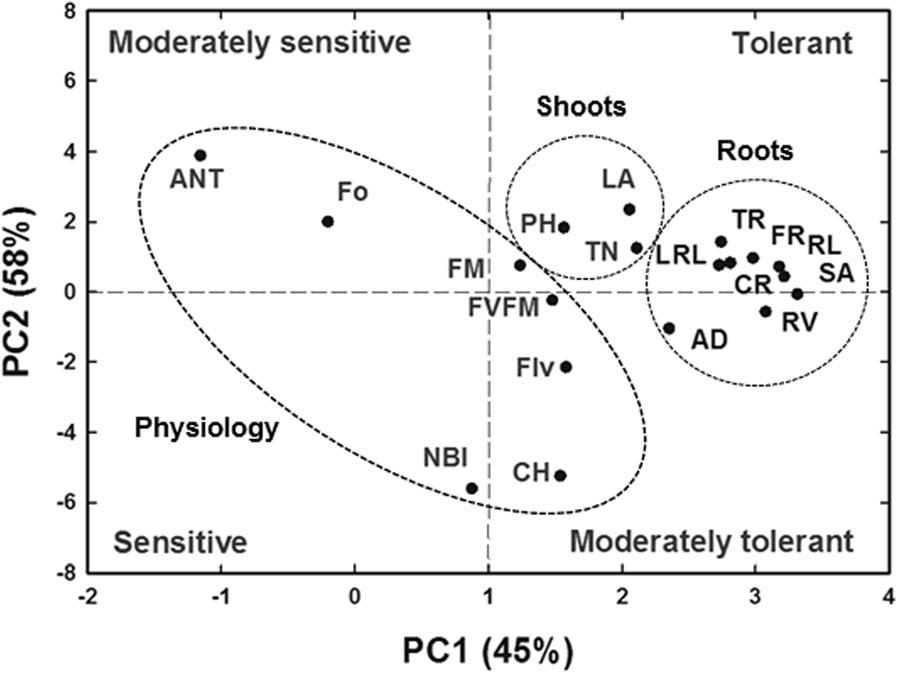
Principal component analysis (PCA) for the first two principal components (PC) scores, PCA1 vs. PCA2 describing the classification salt response parameters measured 37 days after sowing for all the genotypes; plant height (PH), tillers number (TN), leaf area (LA), longest root length (LRL), total root length (TRL), root surface area (SA), average diameter (AD), root volume (RV), root tips (TP), forks (FR), crossings (CR), chlorophyll (CH), flavonoids (FLV), anthocyanin (ANT), and nitrogen balance index (NBI)

PCA was performed to identify the principal components of shoot and root morpho-physiological parameters of rice genotypes that best describe the response to salt stress to identify salt tolerant genotypes. The first two principal components (PCs) accounted for 45% and 13% of the total variation (58%) among rice genotypes, respectively (Fig. 7) and clustered most of the root traits as the best descriptors followed by shoot traits and then physiological traits. The first principal component (PC1) represented higher values for all root parameters and some shoot parameters including total dry weight (TW), TN, and LA, but lesser loadings for all the physiological parameters. The second principal component (PC2) showed higher values for ANT, LA, FO, PH, TP, and LRL, and lesser loadings for NBI, CHL, FLV, AD, RV, FvFm and SA.

**Fig. 8:**
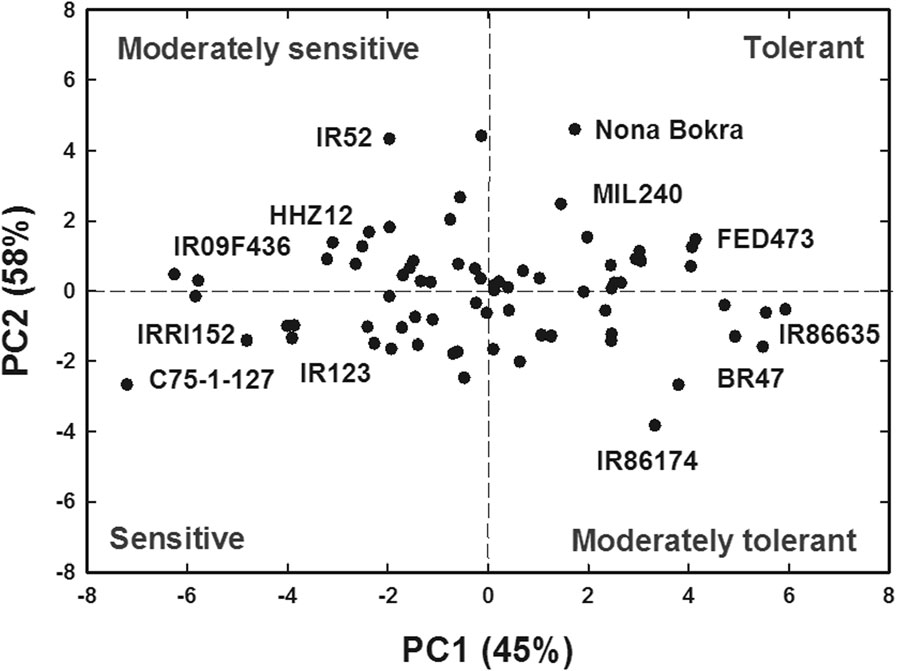
Principal component analysis (PCA) for the first two principal components (PC) scores, PCA1 vs. PCA2 describing the classification of rice genotypes into different salt tolerant groups (salt sensitive, low, moderate, and high salt tolerant) based on all the morpho-physiological parameters measured 37 days after sowing for all the genotypes. Rice genotypes with high/low scores in each tolerant category are identified in the PCA plots

A biplot of PC1 vs. PC2 (Fig. 8) separated the genotypes into different salinity resistance categories. Genotypes showing highest values for the measured shoot and root morpho-physiological parameters for PC1 and PC2, located in the upper-right corner of the biplot, were considered as highly salt tolerant genotypes. Genotypes with moderate values for PC1 and PC2, located in the lower right and upper left corner of the graph, were considered as moderately salt tolerant. In contrast, genotypes showing the low values of the measured shoot and root morpho-physiological parameters for PC1 and PC2 fall in the lower left a portion of the graph and were considered as salt sensitive. Because PC1 and PC2 collectively explained more than half (58%) of the variation and contributed greater importance in the separation of genotypes into different categories, they were used to classify the 74 rice genotypes into four major groups including salt sensitive (19 genotypes, 25.68%), low salt tolerant (20 genotypes, 27.03%), moderately salt tolerant (16 genotypes, 21.62%) and highly salt tolerant (19 genotypes, 25.68%) (Table 3).

**Table 3:** Classification of 74 rice genotypes based on the principal component analysis (PC1 vs. PC2) of morpho-physiological parameters at the seedling stage and the variation accounted by each eigenvector

Salt Sensitive			Low Salt Tolerant			Moderate Salt Tolerant			High Salt Tolerant		
Genotype	PC1	PC2	Genotype	PC1	PC2	Genotype	PC1	PC2	Genotype	PC1	PC2
75–1-127	−7.20747	− 2.66338	IR09F436	−6.25998	0.47198	IR49830	0.10374	−1.6559	CT18244	0.11701	0.16053
IR09L179	−5.83758	−0.15216	IR06N155	−5.7825	0.28962	IR10N230	0.41738	−0.55351	IR86174	0.12364	0.0148
IRRI 152	−4.81707	−1.40919	IR09N537	−3.21539	0.90534	IR70213	0.63453	−2.00713	IR78049	0.21897	0.25885
Rex	−4.00992	−0.99713	HHZ 12	−3.10293	1.37628	IR08N136	1.06702	−1.26436	IR09A130	0.39813	0.09956
IRRI 123	−3.9105	−1.34373	IR10A134	−2.64443	0.76949	CT18247	1.26334	−1.2856	IR93323	0.69812	0.57024
IR86126	−3.86677	−0.98558	IR05N412	−2.5097	1.26958	IR07F287	1.90588	−0.02396	IR04A115	1.0283	0.35985
CT18237	−2.40691	−1.02424	CT18372	−2.37581	1.67961	IR86052	2.34044	−0.56439	MIL 240	1.45441	2.47947
IR6-PAK	−2.27069	−1.49439	IR85427	−1.9708	4.33859	MTU1010	2.45831	−1.42011	N.B	1.73399	4.61212
IR78221	−1.9699	−0.15419	12DS-15	−1.96519	1.8158	FED 2000	2.46614	−1.23379	HHZ 1	1.98	1.52945
COL XXI	−1.93097	−1.64531	IR75483	−1.70066	0.44658	IR86174	3.32968	−3.82995	IRRI 157	2.44828	0.73081
IR65482	−1.71975	−1.04947	CT18615	−1.56465	0.6572	CT18233	3.8009	−2.67039	IR86174	2.46668	0.073
IR07K142	−1.45877	−0.74953	IR09L324	−1.49041	0.84895	12DS-25	4.7199	−0.41126	IR64-NIL	2.51893	0.22771
Apo	−1.40352	−1.53399	FEDE 21	−1.34659	0.2753	BR47	4.92908	−1.29948	IRRI 154	2.65546	0.23095
IR09L337	−1.10877	−0.81638	IR08A172	−1.14798	0.24716	CT18245	5.4841	−1.58231	IR07F102	2.95038	0.91518
FED CARE	−0.69476	−1.78144	IR05F102	−0.75738	2.03509	WAB	5.54291	−0.62458	Geumg	3.02336	1.12504
Thad	−0.61214	−1.73198	CT6946	−0.59829	0.76617	IR86635	5.93269	−0.52805	IR85411	3.05235	0.85483
IR74371	−0.47901	−2.47098	Pokalli	−0.56238	2.6694				PALMAR 18	4.0456	0.70352
CT19561	−0.24034	− 0.34978	IR88633	− 0.26281	0.63098				IR85422	4.06916	1.24965
IR78222	−0.03126	−0.62981	IR65600	−0.14751	0.35194				FED 473	4.13896	1.4758
			CT18614	−0.13627	4.42101						
19			20			16			19		
25.68%			27.03%			21.62%			25.68%		

## Discussion

Salinity tolerance at the seedling stage does not correlate with tolerance at other vegetative and reproductive stages in rice (Ferdose et al. 2009); however, it can adversely affect crop yield by negatively affecting yield-related components (Negrão et al. 2017) including tiller number per plant and shoot biomass (Zeng and Shannon 2000), flowering time (Saade et al. 2016) and harvest index (Gholizadeh et al. 2014). Therefore, crop seedlings need to be well established to increase the ability of the crop to maintain good yield under salinity stress. Early early stage evaluation is, therefore, crucial to identify salt tolerant cultivars with substantial ability to withstand salinity. Since rice is most sensitive to salinity at the seedling or the 2–3 leaf stage (Lutts et al. 1995), it is essential to develop an efficient screening methodology at this early growth stage to identify genotypes possessing genes for salt tolerance. Quijano-Guerta and Kirk (2002) reported that development of a salinity tolerant variety is the cheapest way to address the salinity problem.

Breeding for salinity tolerance in rice involves reliable and rapid screening techniques. Screening under field conditions is difficult due to soil and environmental heterogeneity. These complexities, together with difficulty in creating controlled levels of salinity and reproducibility, make reproducible screening difficult under the field conditions unless multiple environments, years, and replications are used, which increases the cost. IRRI has been using a standard conventional screening in solution culture method at the seedling stage based on visual symptoms of salt stress for mass screening and under controlled conditions to minimize the effect of environmental factors (Gregorio et al. 1997). However, controlled conditions provide a completely different environment to the growing seedlings, which may not perform the same under the field conditions.

Building on IRRI’s simple and rapid screening protocols, we designed a new method to evaluate rice genotypes for salinity tolerance at an early growth stage in pot-culture using pure sand medium, under natural environmental conditions that simulate field conditions. Shoot and root morphological and physiological parameters are measured on all screened rice genotypes; these parameters are more reliable than visual symptoms. Sand growth medium controls soil heterogeneity, which is a significant issue in screening (Negrão et al. 2017).

### Performance of rice genotypes based on shoot and root morpho-physiological parameters and their relationship with salt stress

Salinity negatively affects growth and developmental parameters of rice by reducing shoot length, root length, and plant biomass, which results in the overall decreased growth of the plant (Ali et al. 2014). The decline in growth may be caused by excess toxic NaCl accumulation in the soil around the roots causing imbalanced nutrient uptake by the seedlings. Reduction in leaf area is associated with changes in leaf anatomy due to salinity stress, resulting in a reduced rate of net photosynthesis (Munns and Tester 2008). This may be due to stomatal closure the following internal reduction of CO_2_ and decreased activity of the enzyme RuBisCo (Chaves et al. 2009). Maintenance of photosynthesis is important to maintain normal rates of transpiration under salt stress and is an important indicator of salinity tolerance (Harris et al. 2010). In the current study, salt stress was correlated with a significant reduction in LA in all rice genotypes, potentially contributing to a decline in photosynthesis and a rise in respiration rate in the growing plants. This may have lead to a deficiency of assimilate supply for developing organs and contributed to plant death before maturity. However, disturbance in ion homeostasis can also disturb photosynthesis when Cl- accumulates in the shoot and inhibits photosynthesis (Flowers and Yeo 1988).

Image acquisition technologies are developing rapidly for assessment of plant growth and its response to salinity. For field studies with mature plants, non-destructive approaches with automated high-throughput phenotyping facilities are preferred to evaluate shoot growth (Berger et al. 2012) as they can be used at defined time intervals including before and after salt imposition. Robust, efficient and reliable software is available to analyze and evaluate mature plants in the field, which has been previously used in rice to estimate biomass and relative growth rates (Berger et al. 2012) and growth models (Ward et al. 2015). Similarly, for root evaluation, root imaging has been reported under field conditions using, for example, Growth and Luminescence Observatory (GLO-Roots) system (Rellan-Alvarez et al. 2015), and transparent growth media like gel and glass beads (Courtois et al. 2013). However, root imaging of mature plants is inherently difficult because of the rigorous and hidden root structure (Reynolds et al. 2012). Root imaging is easier at earlier growth stages to characterize and monitor root architecture in response to salinity treatments (Bucksch et al. 2014). Here, we used WinRHIZO imaging with a specialized dual-scan optical scanner and software system to produce high resolution (800 by 800 dpi) gray-scale root images. These were analyzed for eight root parameters.

Past studies have shown that salinity retards plant growth mainly by affecting root growth parameters (Zeng and Shannon 1998; Barua et al. 2015). The increase of root biomass helps tolerant genotypes to maintain vigorous shoot growth possibly through salt dilution or salt exclusion during uptake, limiting the accumulation of the toxic amount of Na + ions in the shoots and resulting in less salinity stress symptoms and more vigorous shoot growth. In the present study, higher root indices indicate greater importance of root parameters than shoot and physiological parameters in identifying salt tolerant rice lines.

### Comparison of PCA and SSRI methods for classification of rice genotypes

Principal component analysis has been previously used to categorize salinity tolerance of canola (*Brassica napus* L., Singh et al. 2008) and corn (*Zea mays* L., Wijewardana et al. 2016). This multidimensional preference analysis allows the identification of parameters that are best described using the tolerance to response variables. It was used here to identify the principal variables that explain the pattern of correlations within the measured salinity stress component traits to identify the parameters best-describing salt tolerance as per (Singh et al. 2008). Principal component analysis can provide an indication and explanation of the crucial component traits contributing to salinity tolerance among the germplasm and conditions under study (Negrão et al. 2017). In the current study, PCA analysis revealed that root, as well as shoot parameters, cluster together, indicating that they are strongly correlated, more so than with physiological parameters.

Salt tolerance indices have been previously used to identify genotypes and parameters with substantial ability to withstand salinity (Munns and James 2003; Genc et al. 2007). Using the total salt stress response indices (TSSRI), we observed well developed and vigorous root systems among salt tolerant genotypes and comparatively less vigorous root systems in salt-sensitive genotypes. Ali et al. (2014) reported that shoot parameters and plant biomass might be better descriptors of salinity tolerance and that root length (the only root parameter they measured) had no significant relation to salinity tolerance. While we also found that some shoot parameters and biomass to be important, Ali et al. (2014) conducted their study in solution culture (NaCl) in glass chambers with a controlled environment, exposing plants to salinity for a shorter duration, and measured too few root parameters compared with the present study and so would not have found the importance of the root traits that we did.

Results of PCA classification of rice genotypes generally agreed with results obtained from the total salt stress response index (TSSRI) method, particularly for the two extreme groups (high salt tolerant and salt sensitive). The intermediate categories (low and moderate salt tolerance) showed slight differences with some genotypes categorized interchangeably. Both PCA and SSRI methods identified root parameters including SA, FR, TRL, RV, CR, LRL, AD, TP to be better descriptors under stress conditions than the shoot traits, indicating the higher importance of root traits in screening rice genotypes for salinity tolerance. SSRI also showed that when salt stress was increased from 6 dSm^− 1^ to 12 dSm^− 1^, the variation explained increased from 62% to 82%. It may be beneficial to screen all genotypes at higher salinity levels and different growth stages, including the flowering stages to find the most salt tolerant genotypes. Similar results between PCA and SSRI support the accuracy of the experiment and the equivalent reliability of the two methods (SSRI and PCA) in screening for stress conditions, including salinity.

## Conclusions

The pot-culture screening technique, designed to control soil heterogeneity and unexpected weather conditions, is a simple and efficient technique for screening rice seedlings for salinity tolerance with a high degree of precision; however, it must be compared with results from future field studies to determine final utility. Although both shoot and root morphological growth and developmental parameters are important indicators of salinity tolerance in rice, this study identified that root parameter are better predictors of salinity tolerance, and physiological parameters are non-predictive. Genotypes which can maintain a deep, well developed and extensive root system will help plants cope under stress conditions by taking up water and nutrients from the soil and efficiently storing them for a longer period for plant survival as compared to genotypes with poorly structured and less vigorous root systems. We also conclude that the two analysis methods (SSRI vs. PCA) are equally reliable and can be used for experiments exclusively and independently, but work better together to confirm the accuracy of experimental results. Knowledge from this study can help rice breeders and other scientists screen and select salinity tolerant rice breeding lines for variety development and related research, and use the lines identified as tolerant in developing new cultivars. This screening method can be used by farmers to screen high yielding commercial cultivars for salinity tolerance at an early stage before taking a potential risk of sowing them in large acreage in salt prone areas.

## Materials and methods

### Experimental conditions and seed material

The experiment was conducted at the Rodney Foil Plant Science Research facility at Mississippi State University, Mississippi State, MS. A total of 74 rice genotypes were obtained from the International Rice Research Institute (IRRI), Philippines (Additional file 1: Table S1) and used with local checks (Thad and Rex) and well-known salt tolerant varieties (Pokali and Nona Bokra) for comparison. PVC pots (15.24 cm diameter, 30.48 cm height, and 5.5 L volume) were arranged in a randomized complete block design (RCBD) with four replications and 74 rice genotypes each. Pots were filled with pure sand (particle size less than 0.3 mm) with 500 g of gravel at the bottom of each pot and grown outdoors to simulate field conditions. Initially, five seeds were sown in each pot which was later thinned to one plant per pot 1 week after seedling emergence. Plants were irrigated three times a day (8 am, 12 pm, and 5 pm) at 90 s per instance through an automated and computer-controlled drip irrigation system.

### Salinity treatment

The three treatments included high salt stress (HSS) with electrical conductivity (EC) of 12 dS/m, medium salt stress (MSS) with EC of 6 dS/m, and control (C) (Hoagland nutrient solution with no additional salts), was imposed 1 week after emergence. In a previous study, Ali et al. (2014) used low (6 dSm^− 1^), moderate (8 and 10 dSm^− 1^) and high (12 and 14 dSm^− 1^) salinity treatments and reported that the low salinity treatment including 6 dSm^− 1^ had no significant effect on seedling morpho-physiological parameters. Therefore, we did not include low salinity treatments in the present study. A mixture of NaCl and CaCl_2_ (5:1 M concentration) was added to full-strength Hoagland nutrient solution (Hewitt 1953), to achieve the final desired EC_w_ of the solutions, which was maintained continuously until harvest. EC_w_ was measured and recorded with an electrical conductivity meter (FieldScout Direct Soil EC Meter, Spectrum Technologies, Aurora, IL, USA) on alternate days by randomly measuring ten pots from each treatment. The pH of the nutrient solution was maintained (using HCl and NaOH solutions) between 5.0 and 6.5 until final harvest (37 days).

### Measurements

#### Root image acquisition analysis

At the final harvest, roots of all plants were cut from the stems and washed on a sieve thoroughly and cautiously to avoid any destruction to the overall root structure. All the roots were then scanned using the WinRHIZO optical scanner (Regent Instruments 2009). First, the 0.3- by 0.2-m Plexiglas tray was filled with approximately 5 mm of tap water, making sure that roots floated in the tray and easily separated with a plastic paint brush to minimize overlapping. The tray was then placed on the top of a specialized dual-scan optical scanner, linked to a computer system. Gray-scale root images were acquired by setting the parameters to high resolution (800 by 800 dpi). Acquired images were analyzed for different root parameters including root surface area (SA), total or cumulative root length (TRL), average root diameter (AD), root volume (RV), number of roots having laterals (RNL), number of tips (TP), number of forks (FR), and number of crossings (CR) using WinRHIZO Pro software.

#### Shoot growth and developmental parameters

Shoot growth and developmental parameters included plant height (PH), tiller number (TN), and leaf area (LA) for all the 74 rice genotypes. Plant height (PH), tiller number (TN) were measured 1 day before the final harvest, whereas leaf area (LA) was measured at the final harvest using leaf-area meter, (LI-3100 Area Meter, Inc., Lincoln, Nebraska, USA). Leaves and stems were then stored separately in the oven at 75 °C for at least 72 h, and leaf dry weight (LW), stem dry weight (SW) and total dry weight (TD) were measured after they were permanently dried.

#### Physiological parameters

Physiological parameters including chlorophyll contents (CH), flavonoids (FLV), anthocyanins (ANT) and nitrogen balance index (NBI), were calculated on-site non-destructively using instruments like soil and plant analyzer (SPAD) meter (SPAD 502 Minnilota Inc. Canada) and Fluropen (Photosystem Instrument Kolackova Czech Republic). SPAD meter was used for instant chlorophyll measurements for all rice genotypes. Similarly, fluorescence including minimal fluorescence intensity (F_o_), maximal fluorescence intensity (F_m_), maximal variable fluorescence (F_v_), and maximum quantum efficiency or yield (F_v_/F_m_) were also measured on-site non-destructively using Fluropen 1000.

### Data analysis

#### Salt stress response indices (SSRI)

Rice genotypes selected for this study were classified into different groups based on their responses to salt stress and subsequent summation of individual index values for each parameter (Raman et al. 2012). Individual salt stress response index (ISSRI) for moderate salt stress was calculated as the value of a parameter (*Pm*) at moderate salt stress for a given genotype divided by the value of the same parameter (*Pc*) at optimum condition (control) (Eq. 1). Similarly, ISSRI for high salt stress was also calculated as the value of a parameter (*Ph*) at high salt stress for a given genotype divided by the value of the same parameter (*Pc*) at optimum condition (control) (Eq. 2).

1 $$ \mathbf{ISSRI}\ \left(\mathbf{moderate}\right)=\mathrm{Pm}/\mathrm{Pc} $$

2 $$ \mathbf{ISSRI}\ \left(\mathbf{high}\right)=\mathrm{Ph}/\mathrm{Pc} $$

The combined or cumulative moderate salt stress response indices (CMSSRI) and combined or cumulative high salt stress response indices (CHSSRI) were calculated by adding all the individual ISSRI for all the 20 measured parameters at moderate (Eq. 3) and high salt stress (Eq. 4), respectively.

3 $$ \mathbf{CMSSRI}=\left(\mathrm{PHm}/\mathrm{PHc}\right)+\left(\mathrm{TNm}/\mathrm{TNc}\right)+\left(\mathrm{LAm}/\mathrm{LAc}\right)+\left(\mathrm{LWm}/\mathrm{LWc}\right)+\left(\mathrm{SWm}/\mathrm{SWc}\right)+\left(\mathrm{RWm}/\mathrm{RWc}\right)+\left(\mathrm{TWm}/\mathrm{TWc}\right)+\left(\mathrm{LRLm}/\mathrm{LRLc}\right)+\left(\mathrm{F}0\mathrm{m}/\mathrm{F}0\mathrm{c}\right)+\left(\mathrm{F}\mathrm{Mm}/\mathrm{F}\mathrm{Mc}\right)+\left(\mathrm{F}\mathrm{Vm}/\mathrm{F}\mathrm{Vc}\right)+\left(\mathrm{F}\mathrm{v}/\mathrm{F}\mathrm{m}\mathrm{m}/\mathrm{F}\mathrm{v}/\mathrm{F}\mathrm{m}\mathrm{c}\right)+\left(\mathrm{TRLm}/\mathrm{TRLc}\right)+\left(\mathrm{SAm}/\mathrm{SAc}\right)+\left(\mathrm{ADm}/\mathrm{ADc}\right)+\left(\mathrm{RVm}/\mathrm{RVc}\right)+\left(\mathrm{RNm}/\mathrm{RNc}\right)+\left(\mathrm{TPm}/\mathrm{TPc}\right)+\left(\mathrm{F}\mathrm{R}\ \mathrm{m}/\mathrm{F}\mathrm{R}\mathrm{c}\right)+\left(\mathrm{CRm}/\mathrm{CRc}\right). $$

4 $$ \mathbf{CHSSRI}=\left(\mathrm{PHh}/\mathrm{PHc}\right)+\left(\mathrm{TNh}/\mathrm{TNc}\right)+\left(\mathrm{LAh}/\mathrm{LAc}\right)+\left(\mathrm{LWh}/\mathrm{LWc}\right)+\left(\mathrm{SWh}/\mathrm{SWc}\right)+\left(\mathrm{RWh}/\mathrm{RWc}\right)+\left(\mathrm{TWh}/\mathrm{TWc}\right)+\left(\mathrm{LRLh}/\mathrm{LRLc}\right)+\left(\mathrm{F}0\mathrm{h}/\mathrm{F}0\right)+\left(\mathrm{F}\mathrm{Mh}/\mathrm{F}\mathrm{Mc}\right)+\left(\mathrm{F}\mathrm{Vh}/\mathrm{F}\mathrm{Vc}\right)+\left(\mathrm{F}\mathrm{v}/\mathrm{F}\mathrm{mh}/\mathrm{F}\mathrm{v}/\mathrm{F}\mathrm{mc}\right)+\left(\mathrm{TRLh}/\mathrm{TRLc}\right)+\left(\mathrm{SAh}/\mathrm{SAc}\right)+\left(\mathrm{ADh}/\mathrm{ADc}\right)+\left(\mathrm{RVh}/\mathrm{RVc}\right)+\left(\mathrm{RNh}/\mathrm{RNc}\right)+\left(\mathrm{TPh}/\mathrm{TPc}\right)+\left(\mathrm{F}\mathrm{Rh}/\mathrm{F}\mathrm{Rc}\right)+\left(\mathrm{CRh}/\mathrm{CRc}\right). $$

Where “c” stands for control, “m” for moderate and “h” for high levels of salinity. Total salt stress response index (TSSRI) (Eq. 5) was calculated as the sum of CMSSRI and CHSSRI (Eq. 3) and (4), respectively.

5 $$ \left(\mathbf{TSSRI}\right)=\mathrm{CMSSRI}+\mathrm{CHSSRI} $$

Finally, based on the TSSRI values of all the measured parameters and standard deviations, the 74 rice genotypes were classified into four response groups including salt sensitive (minimum TSSRI + 0.5SD), low salt tolerant (minimum TSSRI + 1.5SD), moderate salt tolerant (minimum TSSRI + 2.5SD) and high salt tolerant (minimum TSSRI + 3.5SD) genotypes.

#### Statistical analysis

Means, standard deviations (SD), coefficient of variance (CV), and analysis of variance (ANOVA) were calculated using the SAS statistical program (v 9.4, SAS Institute 2011) for all parameters to determine the significant effects (*P* < 0.05) of genotypes, salinity treatment, and their interaction as primary sources of variation. Data were analyzed as a randomized completed block design (RCBD) under two factors arrangement, with genotypes as the main factor and salinity as a sub-main factor. Data were also analyzed via one-way ANOVA using PROC GLM in SAS to determine the effect of salt stress on developmental, physiological, and root parameters. The Fisher’s protected least significance difference test at *P* ≤ 0.05 was employed to test the differences among the treatments for the measured parameters. The standard errors of the means were calculated using Sigma Plot 13.0 (Systat Software, Inc., San Jose, CA, 2008) and presented in the figures as error bars.

Principal component analysis (PCA) was performed on the correlation matrix of 74 rice genotypes and response variables including PH, LA, TN, LW, SW, RW, TW, LRL, TRL, SA, AD, RV, TP, FR, CR, FO, FM, FvFm, CHL, FLV, ANT, NBI. Initially, index values for each treatment were calculated by assessing the response of each shoot, root, and physiological parameter compared to its control value. Then, the responses of all the traits under each treatment were combined and used as index values for PCA analysis. These index values were used to identify the correlation of response variable vectors and genotypes across the ordination space. The analysis was performed using the PRINCOMP procedure in SAS, and results were summarized in biplots (plots of mean PC scores for the first two principal components) using SigmaPlot 13.

## Additional file

Additional file 1: **Table S1.** List of rice genotypes used in this study with accession number, genotype, abbreviated, and country of origin. **Table S2.** Plant height, tillers number, and leaf area of 74 rice genotypes measured 37 days after sowing for control (C), moderate salt stress (MSS) and high salt stress (HSS). Each value represents the mean of four replications. **Table S3.** Total root length, longest root length, root surface area, average diameter, and root volume of 74 rice genotypes under control (C), moderate salt stress (MSS) and high salt stress (HSS) measured 37 days after sowing. Each value represents the mean of four replications. **Table S4.** Root tips, forks, and crossings of 74 rice genotypes under control (C), moderate salt stress (MSS) and high salt stress (HSS), measured 37 days after sowing. Each value represents the mean of four replications. **Table S5.** Chlorophyll, flavonoids, anthocyanin, and nitrogen balance index of 74 rice genotypes under control (C), moderate salt stress (MSS) and high salt stress (HSS), measured 37 days after sowing. Each value represents the mean of four replications. (DOCX 72 kb)

## Data Availability

The datasets generated during and analyzed during the current study are used in the manuscript directly or presented in the supplementary Tables available online.

## Abbreviations

AD: Average root diameter
ANT: Anthocyanin
CHL: Chlorophyll
CR: Root crossings number
FLV: Flavonoids
FM: Maximal fluorescence intensity
FO: Minimal fluorescence intensity,
FR: Root forks number
FvFm: Quantum efficiency of fluorescence
LA: Leaf area
LRL: Longest root length
LW: Leaf dry weight
NBI: Nitrogen balance index
PH: Plant height
RV: Root volume
RW: Root dry weight
SA: Root surface area
SW: Stem dry weight
TN: Tillers number
TP: Root tips number
TRL: Total root length
TW: Total dry weight
