# Supplementary material for: Evaluating rice for salinity using pot-culture provides a systematic tolerance assessment at the seedling stage
Source: Rice (N Y). 2019 Jul 30;12:57. doi: 10.1186/s12284-019-0317-7 (PMC6667605; doi:10.1186/s12284-019-0317-7)
Supplement: Supplementary file 1 — Table S1. List of rice genotypes used in this study with accession number, genotype, abbreviated, and country of origin. Table S2. Plant height, tillers number, and leaf area of 74 rice genotypes measured 37 days after sowing for control (C), moderate salt stress (MSS) and high salt stress (HSS). Each value represents the mean of four replications. Table S3. Total root length, longest root length, root surface area, average diameter, and root volume of 74 rice genotypes under control (C), moderate salt stress (MSS) and high salt stress (HSS) measured 37 days after sowing. Each value represents the mean of four replications. Table S4. Root tips, forks, and crossings of 74 rice genotypes under control (C), moderate salt stress (MSS) and high salt stress (HSS), measured 37 days after sowing. Each value represents the mean of four replications. Table S5. Chlorophyll, flavonoids, anthocyanin, and nitrogen balance index of 74 rice genotypes under control (C), moderate salt stress (MSS) and high salt stress (HSS), measured 37 days after sowing. Each value represents the mean of four replications. (DOCX 72 kb) [file 12284_2019_317_MOESM1_ESM.docx]

Table S1. List of rice genotypes used in this study with accession number, genotype, abbreviated, and country of origin.

| Serial no. | Accession no. | Genotypes | Short Names | Origin |
| --- | --- | --- | --- | --- |
| 1 | 49401 | 12DS-GMET-15 | 12DS-15 | Philippines |
| 2 | 49402 | 12DS-GMET-25 | 12DS-25 | Philippines |
| 3 | 49403 | 75-1-127 | 75-1-127 | Philippines |
| 4 | 49404 | Apo | Apo | Philippines |
| 5 | 49405 | BR47 | BR47 | Philippines |
| 6 | 49406 | COLOMBIA XXI | COL XXI | Philippines |
| 7 | 49407 | CT18233-15-6-6-4-8-1 | CT18233 | Philippines |
| 8 | 49408 | CT18237-13-11-3-3-5-1 | CT18237 | Philippines |
| 9 | 49409 | CT18244-9-4-4-2-1-2 | CT18244 | Philippines |
| 10 | 49410 | CT18245-4-7-1-1-2-1 | CT18245 | Philippines |
| 11 | 49411 | CT18247-12-8-1-4-2-2 | CT18247 | Philippines |
| 12 | 49412 | CT18372-8-1-6-3-1-5 | CT18372 | Philippines |
| 13 | 49413 | Nona Bokra | NB | Philippines |
| 14 | 49414 | CT18614-4-1-2-3-2 | CT18614 | Philippines |
| 15 | 49415 | CT18615-1-5-1-2-1 | CT18615 | Philippines |
| 16 | 49416 | CT19561-3-57-2P-2-1-2-M | CT19561 | Philippines |
| 17 | 49417 | Pokalli | Pokalli | Philippines |
| 18 | 49418 | CT6946-9-1-2-M-1P | CT6946 | Philippines |
| 19 | 49419 | FEDEARROZ 2000 | FED 2000 | Philippines |
| 20 | 49420 | FEDEARROZ 21 | FEDE 21 | Philippines |
| 21 | 49421 | FEDEARROZ 473 | FED 473 | Philippines |
| 22 | 49422 | FEDEARROZ MOCARE | FED CARE | Philippines |
| 23 | 49423 | HHZ 12-DT 10-SAL 1-DT 1 | HHZ 12 | Philippines |
| 24 | 49424 | HHZ 1-Y4-Y1 | HHZ 1 | Philippines |
| 25 | 49425 | IR04A115 | IR04A115 | Philippines |
| 26 | 49426 | IR05F102 | IR05F102 | Philippines |
| 27 | 49427 | IR05N412 | IR05N412 | Philippines |
| 28 | 49428 | IR06N155 | IR06N155 | Philippines |
| 29 | 49429 | IR07F102 | IR07F102 | Philippines |
| 30 | 49430 | IR07F287 | IR07F287 | Philippines |
| 31 | 49431 | IR07K142 | IR07K142 | Philippines |
| 32 | 49432 | IR08A172 | IR08A172 | Philippines |
| 33 | 49433 | IR08N136 | IR08N136 | Philippines |
| 34 | 49434 | IR09A130 | IR09A130 | Philippines |
| 35 | 49435 | IR09F436 | IR09F436 | Philippines |
| 36 | 49436 | IR09L179 | IR09L179 | Philippines |
| 37 | 49437 | IR09L324 | IR09L324 | Philippines |
| 38 | 49438 | IR09L337 | IR09L337 | Philippines |
| 39 | 49439 | IR09N537 | IR09N537 | Philippines |
| 40 | 49440 | IR10A134 | IR10A134 | Philippines |
| 41 | 49441 | IR10N230 | IR10N230 | Philippines |
| 42 | 49442 | IR49830-7-1-2-2 | IR49830 | Philippines |
| 43 | 49443 | IR6 (PAKISTAN) | IR6-PAK | Philippines |
| 44 | 49444 | IR64-EMF NIL | IR64-NIL | Philippines |
| 45 | 49446 | IR65482-4-136-2-2 | IR65482 | Philippines |
| 46 | 49447 | IR65600-81-5-2-3 | IR65600 | Philippines |
| 47 | 49448 | IR70213-10-CPA 4-2-2-2 | IR70213 | Philippines |
| 48 | 49449 | IR74371-70-1-1 | IR74371 | Philippines |
| 49 | 49450 | IR75483-385-2-2 | IR75483 | Philippines |
| 50 | 49451 | IR78049-25-2-2-2 | IR78049 | Philippines |
| 51 | 49452 | IR78221-19-6-33-B-B | IR78221 | Philippines |
| 52 | 49453 | IR78222-20-7-148-2-B | IR78222 | Philippines |
| 53 | 49454 | IR85411 | IR85411 | Philippines |
| 54 | 49456 | IR85422 | IR85422 | Philippines |
| 55 | 49457 | IR85427 | IR85427 | Philippines |
| 56 | 49458 | IR86052-32-3-2 | IR86052 | Philippines |
| 57 | 49459 | IR86126-104-B-B | IR86126 | Philippines |
| 58 | 49460 | IR86174-17-15-2-11-1 | IR86174 | Philippines |
| 59 | 49461 | IR86174-17-15-5-1-28-44 | IR86174 | Philippines |
| 60 | 49462 | IR86174-17-15-B-29-11 | IR86174 | Philippines |
| 61 | 49463 | IR86635-2-3-3-3 | IR86635 | Philippines |
| 62 | 49464 | IR88633:1-66-B-1-B | IR88633 | Philippines |
| 63 | 49465 | IR93323 | IR93323 | Philippines |
| 64 | 49466 | Geumgangbyeo (PI464588) | Geumg | Philippines |
| 65 | 49467 | IRRI 123 | IRRI 123 | Philippines |
| 66 | 49468 | IRRI 152 | IRRI 152 | Philippines |
| 67 | 49469 | IRRI 154 | IRRI 154 | Philippines |
| 68 | 49470 | IRRI 157 | IRRI 157 | Philippines |
| 69 | 49471 | MILYANG 240 | MIL 240 | Philippines |
| 70 | 49472 | MTU1010 | MTU1010 | Philippines |
| 71 | 49474 | PALMAR 18 | PALMAR | Philippines |
| 72 | 49475 | WAB 56-125 | WAB | Philippines |
| 73 | Local check | Thad | Thad | USA |
| 74 | Local check | Rex | Rex | USA |

Table S2. Plant height, tillers number, and leaf area of 74 rice genotypes measured 37 days after sowing for control (C), moderate salt stress (MSS) and high salt stress (HSS). Each value represents the mean of four replications.

|  | Shoot growth and developmental Parameters | | | | | | | | | |
| --- | --- | --- | --- | --- | --- | --- | --- | --- | --- | --- |
|  |  | Plant height | | | Tillers | | | Leaf area | | |
|  |  | cm plant^-1^ | | | no. plant^-1^ | | | cm^-2^ plant^-1^ | | |
| S.No | Genotypes | C MSS HSS | | | C MSS HSS | | | C MSS HSS | | |
| 1 | 12DS-15 | 18.1 | 16.3 | 11.0 | 12.0 | 7.7 | 3.5 | 685.0 | 489.1 | 109.7 |
| 2 | 12DS-25 | 18.3 | 14.3 | 11.0 | 15.3 | 7.2 | 5.3 | 1224.9 | 415.8 | 378.0 |
| 3 | 75-1-127 | 14.5 | 10.0 | 6.0 | 7.0 | 2.3 | 1.3 | 237.1 | 287.9 | 13.5 |
| 4 | Apo | 17.5 | 14.0 | 7.5 | 13.0 | 6.8 | 3.8 | 703.2 | 405.4 | 147.8 |
| 5 | BR47 | 17.5 | 13.7 | 11.2 | 16.5 | 5.8 | 4.8 | 733.8 | 398.6 | 170.3 |
| 6 | COL XXI | 16.5 | 13.3 | 9.8 | 12.0 | 8.1 | 4.0 | 815.8 | 394.8 | 157.7 |
| 7 | CT18233 | 18.5 | 15.3 | 11.2 | 11.3 | 7.8 | 5.4 | 649.5 | 413.6 | 164.5 |
| 8 | CT18237 | 16.5 | 12.2 | 9.9 | 12.0 | 5.7 | 4.1 | 883.3 | 325.5 | 159.3 |
| 9 | CT18244 | 19.5 | 16.7 | 11.4 | 14.8 | 6.7 | 4.3 | 1096.9 | 352.6 | 183.1 |
| 10 | CT18245 | 19.8 | 17.0 | 13.9 | 10.8 | 7.0 | 6.1 | 368.1 | 476.2 | 353.6 |
| 11 | CT18247 | 18.5 | 14.5 | 9.8 | 12.3 | 7.3 | 4.8 | 576.7 | 385.9 | 160.4 |
| 12 | CT18372 | 16.8 | 10.6 | 8.7 | 14.0 | 6.7 | 3.5 | 917.9 | 527.8 | 147.8 |
| 13 | NB | 22.1 | 18.9 | 16.7 | 12.0 | 9.5 | 5.2 | 948.2 | 596.5 | 374.8 |
| 14 | CT18614 | 17.3 | 12.1 | 9.3 | 10.5 | 6.2 | 3.8 | 619.6 | 259.7 | 560.7 |
| 15 | CT18615 | 20.3 | 16.8 | 11.3 | 11.3 | 6.0 | 3.3 | 620.4 | 404.2 | 116.7 |
| 16 | CT19561 | 17.3 | 12.8 | 10.3 | 13.5 | 6.3 | 5.0 | 580.8 | 400.2 | 152.8 |
| 17 | Pokalli | 26.5 | 17.7 | 14.5 | 14.3 | 8.3 | 3.7 | 890.8 | 632.4 | 217.7 |
| 18 | CT6946 | 17.0 | 14.8 | 10.7 | 8.5 | 5.9 | 3.7 | 754.3 | 249.2 | 115.7 |
| 19 | FED 2000 | 17.3 | 12.4 | 11.4 | 15.3 | 8.5 | 4.8 | 692.1 | 341.8 | 130.6 |
| 20 | FEDE 21 | 17.0 | 14.8 | 10.4 | 12.5 | 6.0 | 4.3 | 517.6 | 333.9 | 95.6 |
| 21 | FED 473 | 15.5 | 12.4 | 10.6 | 15.0 | 7.3 | 6.5 | 735.8 | 470.4 | 191.1 |
| 22 | FED CARE | 19.3 | 16.7 | 10.3 | 12.3 | 8.8 | 4.3 | 1054.9 | 375.5 | 121.5 |
| 23 | HHZ 12 | 21.5 | 16.9 | 9.0 | 9.8 | 6.0 | 3.0 | 405.7 | 246.0 | 80.8 |
| 24 | HHZ 1 | 15.0 | 12.4 | 9.9 | 11.0 | 6.8 | 5.5 | 783.1 | 346.0 | 159.2 |
| 25 | IR04A115 | 18.5 | 14.1 | 11.8 | 13.0 | 6.3 | 3.8 | 769.0 | 245.3 | 153.0 |
| 26 | IR05F102 | 18.0 | 15.0 | 10.8 | 11.8 | 6.1 | 3.8 | 592.3 | 299.1 | 154.4 |
| 27 | IR05N412 | 15.3 | 10.2 | 5.8 | 14.3 | 7.0 | 4.8 | 872.6 | 365.7 | 160.9 |
| 28 | IR06N155 | 16.5 | 12.4 | 8.7 | 10.8 | 4.8 | 1.8 | 352.4 | 255.8 | 176.9 |
| 29 | IR07F102 | 19.5 | 16.3 | 12.8 | 12.0 | 8.3 | 5.7 | 902.1 | 440.4 | 216.7 |
| 30 | IR07F287 | 20.4 | 16.3 | 13.8 | 19.8 | 8.1 | 7.3 | 1071.2 | 471.2 | 277.1 |
| 31 | IR07K142 | 20.5 | 16.0 | 10.8 | 16.3 | 7.3 | 4.7 | 858.4 | 542.8 | 177.0 |
| 32 | IR08A172 | 19.8 | 16.3 | 10.2 | 15.0 | 7.8 | 4.8 | 679.6 | 403.3 | 201.0 |
| 33 | IR08N136 | 19.5 | 14.7 | 11.2 | 11.5 | 7.7 | 4.7 | 646.5 | 342.7 | 137.3 |
| 34 | IR09A130 | 19.3 | 14.3 | 11.3 | 16.0 | 6.8 | 5.4 | 1005.5 | 418.4 | 192.0 |
| 35 | IR09F436 | 16.8 | 14.7 | 10.2 | 5.3 | 3.3 | 1.8 | 354.5 | 203.8 | 41.5 |
| 36 | IR09L179 | 17.8 | 13.6 | 10.0 | 4.8 | 3.3 | 1.8 | 271.4 | 105.5 | 40.1 |
| 37 | IR09L324 | 19.5 | 16.4 | 13.2 | 13.0 | 7.7 | 4.8 | 918.9 | 306.7 | 192.6 |
| 38 | IR09L337 | 15.5 | 14.3 | 10.5 | 8.0 | 4.7 | 4.0 | 288.6 | 279.6 | 71.4 |
| 39 | IR09N537 | 19.4 | 12.7 | 8.8 | 11.0 | 5.7 | 2.8 | 584.7 | 255.7 | 179.0 |
| 40 | IR10A134 | 21.0 | 16.3 | 11.5 | 3.8 | 3.2 | 1.7 | 185.8 | 228.9 | 134.7 |
| 41 | IR10N230 | 16.3 | 12.5 | 8.9 | 15.5 | 8.8 | 5.0 | 947.5 | 424.7 | 160.7 |
| 42 | IR49830 | 20.3 | 13.8 | 12.2 | 7.5 | 6.1 | 3.9 | 513.8 | 315.4 | 104.4 |
| 43 | IR6-PAK | 14.0 | 9.7 | 7.3 | 10.3 | 6.4 | 2.7 | 813.8 | 492.3 | 89.8 |
| 44 | IR64-NIL | 18.5 | 14.8 | 12.9 | 11.0 | 7.8 | 4.3 | 513.7 | 312.9 | 269.0 |
| 45 | IR65482 | 15.8 | 13.6 | 10.7 | 11.0 | 5.3 | 3.5 | 632.3 | 374.1 | 108.6 |
| 46 | IR65600 | 16.0 | 12.1 | 10.8 | 12.5 | 6.3 | 4.4 | 751.8 | 289.4 | 194.3 |
| 47 | IR70213 | 13.0 | 13.3 | 8.8 | 6.5 | 5.4 | 3.3 | 832.9 | 279.6 | 91.7 |
| 48 | IR74371 | 18.3 | 12.7 | 10.7 | 9.3 | 6.0 | 3.8 | 630.3 | 252.8 | 115.7 |
| 49 | IR75483 | 16.5 | 13.0 | 9.3 | 11.0 | 5.8 | 4.3 | 792.0 | 246.2 | 85.6 |
| 50 | IR78049 | 19.0 | 14.7 | 9.9 | 12.3 | 7.3 | 3.8 | 1148.8 | 404.0 | 196.0 |
| 51 | IR78221 | 18.8 | 13.8 | 11.6 | 12.0 | 5.7 | 3.7 | 688.2 | 271.6 | 92.4 |
| 52 | IR78222 | 19.8 | 15.7 | 10.0 | 13.0 | 6.0 | 5.7 | 752.7 | 251.1 | 128.9 |
| 53 | IR85411 | 18.5 | 14.0 | 13.1 | 15.0 | 6.8 | 7.3 | 644.4 | 341.6 | 199.0 |
| 54 | IR85422 | 20.0 | 15.4 | 12.8 | 13.8 | 6.3 | 5.1 | 914.6 | 408.3 | 315.6 |
| 55 | IR85427 | 24.3 | 17.8 | 16.0 | 9.5 | 5.3 | 3.7 | 909.2 | 306.4 | 153.1 |
| 56 | IR86052 | 30.3 | 22.7 | 19.3 | 9.3 | 4.3 | 4.0 | 501.6 | 257.7 | 195.9 |
| 57 | IR86126 | 17.1 | 14.0 | 10.3 | 11.0 | 3.7 | 2.3 | 496.8 | 387.6 | 151.7 |
| 58 | IR86174 | 14.5 | 11.7 | 9.7 | 15.3 | 6.7 | 4.3 | 917.2 | 348.1 | 211.5 |
| 59 | IR86174 | 20.3 | 17.0 | 12.3 | 16.0 | 7.7 | 5.4 | 629.4 | 365.0 | 243.5 |
| 60 | IR86174 | 19.8 | 15.5 | 11.4 | 7.5 | 5.7 | 4.0 | 752.7 | 364.4 | 196.3 |
| 61 | IR86635 | 17.1 | 14.7 | 10.7 | 17.3 | 10.7 | 7.0 | 1427.3 | 879.2 | 432.2 |
| 62 | IR88633 | 17.2 | 13.4 | 8.9 | 16.0 | 8.7 | 4.3 | 938.4 | 496.2 | 171.8 |
| 63 | IR93323 | 20.3 | 15.8 | 12.6 | 11.0 | 5.2 | 4.6 | 699.8 | 180.0 | 216.0 |
| 64 | Geumg | 14.8 | 10.8 | 8.6 | 15.0 | 5.8 | 5.7 | 493.9 | 243.7 | 142.0 |
| 65 | IRRI 123 | 15.3 | 8.3 | 6.1 | 12.0 | 5.3 | 3.8 | 609.9 | 159.2 | 48.5 |
| 66 | IRRI 152 | 17.3 | 16.0 | 9.1 | 12.8 | 6.3 | 2.3 | 320.2 | 326.6 | 175.1 |
| 67 | IRRI 154 | 16.3 | 14.3 | 10.0 | 14.5 | 10.1 | 5.8 | 760.4 | 455.5 | 252.1 |
| 68 | IRRI 157 | 24.9 | 20.3 | 16.7 | 13.5 | 6.3 | 3.7 | 1418.3 | 342.5 | 207.6 |
| 69 | MIL 240 | 16.4 | 14.8 | 12.2 | 10.0 | 8.3 | 4.3 | 650.8 | 424.5 | 195.7 |
| 70 | MTU1010 | 18.3 | 12.3 | 10.6 | 11.3 | 7.3 | 4.6 | 838.1 | 424.9 | 179.3 |
| 71 | PALMAR | 19.0 | 16.3 | 12.3 | 11.5 | 6.1 | 4.7 | 637.2 | 287.3 | 232.3 |
| 72 | WAB | 16.8 | 12.8 | 14.3 | 13.5 | 5.7 | 7.3 | 943.1 | 242.0 | 248.2 |
| 73 | Thad | 19.5 | 15.3 | 14.1 | 7.0 | 4.3 | 3.7 | 455.2 | 151.7 | 76.1 |
| 74 | Rex | 16.7 | 14.8 | 10.5 | 6.5 | 3.3 | 2.3 | 537.3 | 170.5 | 65.1 |
|  | **Means** | **18.3** | **14.4** | **11.0** | **11.9** | **6.5** | **4.3** | **721.1** | **353.7** | **174.5** |

Table S3. Total root length, longest root length, root surface area, average diameter, and root volume of 74 rice genotypes under control (C), moderate salt stress (MSS) and high salt stress (HSS) measured 37 days after sowing. Each value represents the mean of four replications.

| Root growth parameters | | | | | | | | | | | | | | | | |
| --- | --- | --- | --- | --- | --- | --- | --- | --- | --- | --- | --- | --- | --- | --- | --- | --- |
|  |  | Total root length | | | Longest root length | | | Surface area | | | Average diameter | | | Root volume | | |
|  |  | cm plants^-1^ | | | cm, plant^-1^ | | | cm^-2^ plan^-1^ | | | mm, root^-1^ plant^-1^ | | | cm^-3^ plant^-1^ | | |
| S.No | Genotypes | C | MSS | HSS | C | MSS | HSS | C | MSS | HSS | C | MSS | HSS | C | MSS | HSS |
| 1 | 12DS-15 | 5744.5 | 5584.5 | 4316.4 | 38.5 | 45.0 | 33.0 | 1259.0 | 1142.5 | 541.9 | 0.7 | 0.7 | 0.4 | 22.1 | 18.6 | 5.5 |
| 2 | 12DS-25 | 7057.7 | 4901.1 | 6809.4 | 51.8 | 42.8 | 37.0 | 1683.2 | 1201.7 | 1153.9 | 0.8 | 0.8 | 0.5 | 33.1 | 23.5 | 16.2 |
| 3 | 75-1-127 | 4352.5 | 3670.9 | 669.3 | 40.0 | 33.3 | 16.5 | 639.2 | 642.2 | 79.5 | 0.5 | 0.5 | 0.5 | 7.8 | 9.0 | 0.8 |
| 4 | Apo | 6651.4 | 6195.4 | 4361.2 | 43.0 | 47.8 | 26.3 | 1274.4 | 1245.8 | 649.3 | 0.6 | 0.6 | 0.4 | 20.0 | 19.9 | 8.2 |
| 5 | BR47 | 6193.3 | 6093.9 | 7516.7 | 47.0 | 44.3 | 39.8 | 1500.2 | 1060.5 | 1130.5 | 0.8 | 0.6 | 0.5 | 32.1 | 14.9 | 14.2 |
| 6 | COL XXI | 6080.4 | 5768.0 | 4012.5 | 47.3 | 47.5 | 29.5 | 1345.5 | 1390.4 | 451.3 | 0.7 | 0.8 | 0.4 | 24.5 | 26.9 | 4.2 |
| 7 | CT18233 | 4820.0 | 6344.7 | 6586.6 | 39.0 | 46.8 | 37.8 | 942.7 | 1206.7 | 957.1 | 0.6 | 0.6 | 0.5 | 14.8 | 18.5 | 12.0 |
| 8 | CT18237 | 7342.6 | 5648.9 | 3488.7 | 49.8 | 44.8 | 27.8 | 1422.5 | 993.2 | 419.5 | 0.6 | 0.6 | 0.4 | 22.7 | 14.1 | 4.1 |
| 9 | CT18244 | 5683.7 | 6075.0 | 5040.3 | 45.0 | 45.0 | 36.0 | 1341.3 | 1260.8 | 699.3 | 0.7 | 0.7 | 0.4 | 26.1 | 21.2 | 7.9 |
| 10 | CT18245 | 6360.9 | 5538.6 | 6001.7 | 42.3 | 39.5 | 26.8 | 1479.7 | 1172.8 | 1257.4 | 0.7 | 0.7 | 0.6 | 27.6 | 20.4 | 21.3 |
| 11 | CT18247 | 6184.3 | 5707.9 | 5797.7 | 40.5 | 44.3 | 28.8 | 1330.3 | 1112.3 | 734.1 | 0.7 | 0.6 | 0.4 | 23.3 | 18.3 | 7.4 |
| 12 | CT18372 | 6429.9 | 3974.1 | 4339.5 | 45.0 | 35.5 | 32.3 | 1169.1 | 835.1 | 459.1 | 0.6 | 0.6 | 0.4 | 17.1 | 14.2 | 3.9 |
| 13 | NB | 5733.5 | 6271.8 | 5152.7 | 37.3 | 43.3 | 34.0 | 1193.3 | 1366.6 | 815.5 | 0.7 | 0.7 | 0.5 | 19.8 | 24.0 | 10.4 |
| 14 | CT18614 | 5928.4 | 4387.8 | 5198.2 | 47.5 | 40.0 | 37.8 | 1192.3 | 899.2 | 657.3 | 0.7 | 0.7 | 0.4 | 19.8 | 14.7 | 6.7 |
| 15 | CT18615 | 6365.9 | 6222.4 | 4033.6 | 38.8 | 42.0 | 30.0 | 1246.2 | 1239.6 | 465.8 | 0.6 | 0.6 | 0.4 | 20.5 | 19.8 | 4.4 |
| 16 | CT19561 | 6246.1 | 4633.7 | 4694.9 | 42.0 | 38.5 | 30.5 | 1296.2 | 946.1 | 643.4 | 0.7 | 0.7 | 0.4 | 21.5 | 15.5 | 7.5 |
| 17 | Pokalli | 5962.9 | 6256.3 | 4883.8 | 38.5 | 41.8 | 34.3 | 1351.7 | 1349.3 | 650.5 | 0.7 | 0.7 | 0.4 | 26.6 | 23.5 | 7.2 |
| 18 | CT6946 | 6453.5 | 6007.4 | 4950.2 | 48.3 | 41.0 | 38.5 | 1371.8 | 991.2 | 686.8 | 0.7 | 0.5 | 0.4 | 23.6 | 13.1 | 7.8 |
| 19 | FED 2000 | 5571.7 | 5439.4 | 5884.2 | 40.5 | 39.3 | 35.0 | 1325.9 | 1136.1 | 1012.0 | 0.8 | 0.7 | 0.5 | 26.1 | 19.6 | 14.6 |
| 20 | FEDE 21 | 6102.7 | 4983.0 | 4270.0 | 39.8 | 42.8 | 30.5 | 1109.6 | 968.8 | 611.6 | 0.6 | 0.6 | 0.4 | 17.1 | 15.6 | 7.7 |
| 21 | FED 473 | 4621.4 | 6223.3 | 6158.4 | 30.8 | 44.8 | 26.0 | 1030.9 | 1160.6 | 1129.4 | 0.7 | 0.6 | 0.6 | 18.9 | 17.5 | 16.9 |
| 22 | FED CARE | 6431.8 | 6427.2 | 4864.8 | 43.8 | 51.3 | 35.0 | 1529.0 | 1394.3 | 747.5 | 0.8 | 0.7 | 0.4 | 29.3 | 24.7 | 10.0 |
| 23 | HHZ 12 | 6197.1 | 6247.0 | 3288.6 | 47.0 | 42.3 | 34.0 | 1165.4 | 1130.2 | 583.8 | 0.6 | 0.6 | 0.5 | 17.7 | 16.3 | 8.6 |
| 24 | HHZ 1 | 6220.5 | 5645.3 | 6617.1 | 44.0 | 40.5 | 39.8 | 1152.1 | 1049.4 | 865.1 | 0.6 | 0.6 | 0.4 | 18.3 | 16.3 | 9.4 |
| 25 | IR04A115 | 6142.3 | 6094.1 | 5689.2 | 43.5 | 40.8 | 32.5 | 1346.6 | 1119.7 | 725.1 | 0.7 | 0.6 | 0.4 | 24.1 | 16.7 | 7.9 |
| 26 | IR05F102 | 5978.6 | 4595.0 | 5522.4 | 38.8 | 35.3 | 30.8 | 1217.8 | 918.0 | 615.3 | 0.6 | 0.6 | 0.4 | 20.4 | 15.2 | 5.6 |
| 27 | IR05N412 | 5343.9 | 5290.1 | 4206.9 | 36.5 | 34.8 | 27.8 | 1081.1 | 1011.8 | 421.9 | 0.7 | 0.6 | 0.3 | 17.7 | 15.5 | 3.5 |
| 28 | IR06N155 | 3896.7 | 4511.7 | 1549.0 | 37.8 | 42.8 | 21.5 | 597.3 | 578.1 | 164.5 | 0.5 | 0.4 | 0.4 | 7.4 | 6.1 | 1.4 |
| 29 | IR07F102 | 7021.1 | 6698.4 | 5734.1 | 45.8 | 41.5 | 41.0 | 1444.4 | 1262.0 | 951.6 | 0.7 | 0.6 | 0.5 | 24.6 | 19.0 | 14.3 |
| 30 | IR07F287 | 6660.8 | 4783.0 | 4026.5 | 47.0 | 38.3 | 31.0 | 1434.9 | 1052.0 | 756.6 | 0.7 | 0.7 | 0.5 | 25.2 | 18.7 | 12.8 |
| 31 | IR07K142 | 5121.5 | 6285.4 | 3754.8 | 39.5 | 42.0 | 34.3 | 1182.3 | 1374.0 | 449.6 | 0.7 | 0.7 | 0.4 | 22.6 | 23.9 | 4.4 |
| 32 | IR08A172 | 7561.8 | 4991.2 | 4120.1 | 43.0 | 41.8 | 35.3 | 1505.5 | 1370.8 | 477.9 | 0.6 | 0.9 | 0.4 | 24.3 | 30.0 | 4.6 |
| 33 | IR08N136 | 4213.4 | 5958.8 | 5266.0 | 36.3 | 37.3 | 34.0 | 920.5 | 1031.5 | 827.6 | 0.7 | 0.6 | 0.5 | 16.7 | 14.3 | 10.9 |
| 34 | IR09A130 | 5800.8 | 4732.0 | 4805.6 | 40.8 | 40.5 | 35.3 | 1455.5 | 1136.4 | 670.6 | 0.9 | 0.8 | 0.5 | 32.5 | 21.8 | 7.5 |
| 35 | IR09F436 | 4312.4 | 2869.1 | 2034.4 | 31.8 | 32.0 | 13.3 | 707.6 | 393.2 | 184.5 | 0.5 | 0.4 | 0.3 | 9.4 | 4.3 | 1.3 |
| 36 | IR09L179 | 3535.0 | 3881.5 | 2022.2 | 33.5 | 36.3 | 17.0 | 529.3 | 510.2 | 260.4 | 0.5 | 0.4 | 0.4 | 6.3 | 5.4 | 2.7 |
| 37 | IR09L324 | 6083.3 | 5702.8 | 3947.0 | 44.8 | 51.5 | 39.3 | 1366.9 | 1187.2 | 516.1 | 0.8 | 0.7 | 0.4 | 26.5 | 20.4 | 5.4 |
| 38 | IR09L337 | 5012.7 | 2923.7 | 4296.1 | 42.5 | 38.5 | 28.0 | 747.6 | 538.7 | 579.9 | 0.5 | 0.6 | 0.4 | 9.1 | 8.0 | 6.3 |
| 39 | IR09N537 | 6586.3 | 6084.5 | 3302.6 | 46.8 | 37.8 | 30.5 | 1309.3 | 1024.1 | 430.5 | 0.6 | 0.5 | 0.4 | 21.1 | 13.9 | 4.7 |
| 40 | IR10A134 | 3201.3 | 2818.8 | 4332.6 | 32.0 | 33.3 | 17.0 | 469.3 | 368.7 | 619.7 | 0.5 | 0.4 | 0.4 | 5.5 | 3.8 | 7.9 |
| 41 | IR10N230 | 5658.0 | 4802.7 | 4743.7 | 50.3 | 39.8 | 32.3 | 1088.0 | 940.0 | 777.6 | 0.6 | 0.6 | 0.5 | 17.0 | 14.7 | 11.0 |
| 42 | IR49830 | 6725.1 | 4563.7 | 4900.4 | 36.5 | 43.0 | 28.3 | 1103.9 | 777.9 | 718.7 | 0.5 | 0.5 | 0.4 | 14.5 | 10.8 | 9.1 |
| 43 | IR6-PAK | 5716.3 | 4593.9 | 4131.4 | 39.8 | 39.5 | 26.0 | 1176.6 | 1038.0 | 536.4 | 0.7 | 0.7 | 0.4 | 20.4 | 18.7 | 5.8 |
| 44 | IR64-NIL | 7245.7 | 6244.8 | 6329.6 | 50.3 | 40.0 | 37.3 | 1561.1 | 1235.8 | 816.2 | 0.7 | 0.6 | 0.4 | 27.3 | 19.5 | 8.8 |
| 45 | IR65482 | 6079.2 | 4621.9 | 3815.2 | 42.5 | 34.5 | 26.8 | 1406.6 | 787.6 | 528.1 | 0.7 | 0.5 | 0.4 | 26.9 | 10.7 | 5.9 |
| 46 | IR65600 | 5561.9 | 4851.4 | 4250.2 | 43.5 | 40.0 | 32.0 | 1289.3 | 877.4 | 546.0 | 0.7 | 0.6 | 0.4 | 25.5 | 12.8 | 5.6 |
| 47 | IR70213 | 6251.4 | 6011.9 | 5389.0 | 38.8 | 46.3 | 37.3 | 1526.1 | 1019.4 | 738.0 | 0.8 | 0.5 | 0.4 | 31.7 | 13.8 | 8.4 |
| 48 | IR74371 | 7126.2 | 5923.5 | 4424.6 | 48.8 | 45.0 | 23.8 | 1402.8 | 1018.4 | 704.9 | 0.6 | 0.6 | 0.5 | 22.3 | 14.0 | 10.0 |
| 49 | IR75483 | 5498.0 | 5518.2 | 4130.7 | 41.5 | 41.3 | 36.5 | 1138.9 | 830.3 | 474.3 | 0.7 | 0.5 | 0.4 | 20.2 | 10.1 | 4.4 |
| 50 | IR78049 | 6368.4 | 5668.2 | 5298.9 | 44.3 | 42.3 | 42.0 | 1404.5 | 1152.7 | 621.8 | 0.7 | 0.7 | 0.4 | 25.2 | 18.9 | 6.0 |
| 51 | IR78221 | 6083.5 | 4520.4 | 3995.8 | 45.3 | 41.5 | 35.3 | 1504.9 | 933.4 | 482.5 | 0.8 | 0.7 | 0.4 | 31.7 | 15.3 | 4.7 |
| 52 | IR78222 | 6149.5 | 5230.4 | 5017.4 | 46.5 | 41.8 | 35.8 | 1294.6 | 863.9 | 576.6 | 0.7 | 0.5 | 0.4 | 23.7 | 11.6 | 5.5 |
| 53 | IR85411 | 5742.9 | 6060.5 | 6088.7 | 40.3 | 44.0 | 37.5 | 1155.6 | 1301.7 | 893.1 | 0.6 | 0.7 | 0.5 | 18.7 | 22.4 | 11.2 |
| 54 | IR85422 | 6266.3 | 6481.9 | 6733.4 | 46.5 | 37.8 | 43.3 | 1538.5 | 1199.7 | 1102.9 | 0.8 | 0.6 | 0.5 | 33.0 | 17.7 | 14.8 |
| 55 | IR85427 | 6394.6 | 4414.6 | 4131.8 | 43.5 | 35.5 | 39.8 | 1219.8 | 726.7 | 453.0 | 0.6 | 0.5 | 0.4 | 18.6 | 9.6 | 4.0 |
| 56 | IR86052 | 7122.0 | 5181.7 | 5085.5 | 51.8 | 40.3 | 41.3 | 1460.9 | 968.3 | 899.1 | 0.7 | 0.6 | 0.5 | 24.6 | 15.1 | 12.8 |
| 57 | IR86126 | 5685.1 | 3591.2 | 2590.7 | 45.0 | 32.8 | 19.3 | 957.2 | 641.5 | 362.3 | 0.5 | 0.6 | 0.4 | 13.2 | 9.1 | 4.1 |
| 58 | IR86174 | 6643.8 | 6335.7 | 6161.2 | 44.3 | 41.3 | 33.3 | 1471.7 | 1077.4 | 995.7 | 0.7 | 0.5 | 0.5 | 27.7 | 14.9 | 12.9 |
| 59 | IR86174 | 6617.8 | 5099.5 | 5880.1 | 43.5 | 40.8 | 40.0 | 1277.9 | 1094.4 | 857.9 | 0.6 | 0.7 | 0.5 | 21.1 | 18.8 | 10.6 |
| 60 | IR86174 | 5907.1 | 4982.6 | 4613.4 | 39.0 | 39.3 | 40.0 | 1159.0 | 1101.2 | 657.8 | 0.6 | 0.7 | 0.4 | 18.8 | 19.5 | 7.9 |
| 61 | IR86635 | 7355.5 | 5359.3 | 7144.1 | 44.5 | 44.3 | 36.3 | 1732.6 | 1236.9 | 1107.1 | 0.8 | 0.7 | 0.5 | 33.1 | 23.7 | 14.6 |
| 62 | IR88633 | 6493.3 | 5690.9 | 5243.5 | 43.0 | 42.3 | 35.5 | 1394.5 | 1218.8 | 674.3 | 0.7 | 0.7 | 0.4 | 24.3 | 20.8 | 7.2 |
| 63 | IR93323 | 5991.7 | 5293.6 | 4765.0 | 49.0 | 44.8 | 32.8 | 1268.8 | 967.9 | 796.8 | 0.7 | 0.6 | 0.5 | 22.3 | 14.1 | 10.7 |
| 64 | Geumg | 7577.5 | 4988.6 | 7384.3 | 44.3 | 42.0 | 44.8 | 1420.4 | 846.5 | 955.8 | 0.6 | 0.5 | 0.4 | 21.9 | 11.4 | 9.9 |
| 65 | IRRI 123 | 4761.4 | 5179.2 | 2682.2 | 39.0 | 42.0 | 22.0 | 899.1 | 883.4 | 292.2 | 0.6 | 0.5 | 0.4 | 14.2 | 12.1 | 2.5 |
| 66 | IRRI 152 | 4919.9 | 4037.7 | 3142.7 | 40.0 | 41.5 | 24.3 | 1031.4 | 780.4 | 330.5 | 0.7 | 0.6 | 0.3 | 17.6 | 12.2 | 2.8 |
| 67 | IRRI 154 | 5891.3 | 6021.0 | 6625.1 | 44.5 | 40.5 | 29.0 | 1232.1 | 1147.5 | 826.6 | 0.7 | 0.6 | 0.4 | 22.1 | 17.7 | 8.3 |
| 68 | IRRI 157 | 5368.5 | 5156.3 | 5559.4 | 45.8 | 40.5 | 31.0 | 1571.8 | 1225.5 | 858.8 | 0.9 | 0.8 | 0.5 | 37.6 | 23.2 | 11.2 |
| 69 | MIL 240 | 6127.0 | 6168.5 | 6650.8 | 46.5 | 45.8 | 38.8 | 1351.2 | 1224.5 | 775.5 | 0.7 | 0.6 | 0.4 | 24.8 | 19.5 | 7.3 |
| 70 | MTU1010 | 6789.4 | 5356.6 | 5163.0 | 51.3 | 39.0 | 51.5 | 1716.4 | 1213.6 | 825.5 | 0.8 | 0.7 | 0.5 | 37.0 | 22.0 | 10.8 |
| 71 | PALMAR | 6208.8 | 6340.2 | 7503.1 | 53.3 | 41.5 | 40.0 | 1212.1 | 1298.0 | 1090.1 | 0.6 | 0.7 | 0.5 | 19.0 | 21.6 | 13.3 |
| 72 | WAB | 6197.0 | 5558.0 | 6172.0 | 49.5 | 39.3 | 42.8 | 1421.7 | 948.7 | 1104.7 | 0.7 | 0.5 | 0.6 | 26.4 | 12.9 | 17.0 |
| 73 | Thad | 5992.9 | 4861.8 | 4450.8 | 46.3 | 43.5 | 47.3 | 1237.2 | 821.3 | 508.1 | 0.7 | 0.5 | 0.4 | 21.5 | 11.2 | 4.7 |
| 74 | Rex | 5929.6 | 4006.2 | 2775.8 | 45.3 | 45.8 | 32.0 | 961.5 | 723.8 | 301.9 | 0.5 | 0.6 | 0.4 | 12.4 | 10.9 | 2.6 |
|  | **Means** | **5968.3** | **5286.3** | **4817.8** | **43.1** | **41.2** | **33.0** | **1246.7** | **1025.6** | **679.0** | **0.7** | **0.6** | **0.4** | **21.9** | **16.3** | **8.2** |

Table S4. Root tips, forks, and crossings of 74 rice genotypes under control (C), moderate salt stress (MSS) and high salt stress (HSS), measured 37 days after sowing. Each value represents the mean of four replications.

| Root developmental parameters | | | | | | | | | | |
| --- | --- | --- | --- | --- | --- | --- | --- | --- | --- | --- |
|  |  | Tips, no. plant^-1^ | | | Forks, no. plant^-1^ | | | Crossings, no. plant^-1^ | | |
| S.No | Genotypes | C | MSS | HSS | C | MSS | HSS | C | MSS | HSS |
| 1 | 12DS-15 | 32814.7 | 32571.0 | 26673.3 | 111674.7 | 101004.0 | 59984.3 | 7698.0 | 6698.7 | 5088.7 |
| 2 | 12DS-25 | 31776.3 | 24604.7 | 39289.7 | 128486.3 | 81853.7 | 106946.7 | 8308.3 | 4449.3 | 7797.3 |
| 3 | 75-1-127 | 28253.3 | 24395.7 | 5476.0 | 61464.0 | 56241.7 | 6841.3 | 5035.0 | 3600.3 | 649.7 |
| 4 | Apo | 38546.0 | 34908.0 | 26229.0 | 119729.3 | 106635.7 | 62002.3 | 9927.0 | 7392.7 | 4973.3 |
| 5 | BR47 | 33436.0 | 40827.0 | 45566.3 | 129611.3 | 103564.3 | 124605.7 | 9132.7 | 7143.0 | 10991.0 |
| 6 | COL XXI | 32943.7 | 31824.3 | 24202.0 | 110794.0 | 106497.7 | 47972.7 | 7820.3 | 6811.0 | 4628.7 |
| 7 | CT18233 | 31654.7 | 36014.3 | 35365.3 | 88791.0 | 105271.7 | 100965.3 | 6404.3 | 7122.0 | 9353.0 |
| 8 | CT18237 | 39459.0 | 33460.3 | 24764.7 | 132320.0 | 92738.3 | 47819.3 | 9666.0 | 6922.0 | 4166.7 |
| 9 | CT18244 | 30647.7 | 33242.7 | 27136.0 | 107631.0 | 102136.0 | 68197.7 | 7096.3 | 6382.3 | 6115.7 |
| 10 | CT18245 | 35646.0 | 32497.7 | 29389.3 | 121759.7 | 95973.7 | 111642.0 | 8762.0 | 6227.0 | 7667.3 |
| 11 | CT18247 | 31539.3 | 33759.7 | 35841.7 | 106878.0 | 97429.7 | 87168.7 | 7163.7 | 6564.0 | 7989.0 |
| 12 | CT18372 | 37163.7 | 24358.0 | 24907.0 | 116375.7 | 70570.3 | 64253.0 | 8746.7 | 4194.0 | 6847.3 |
| 13 | NB | 32975.3 | 32022.0 | 29429.3 | 103779.0 | 100018.7 | 80902.7 | 7556.7 | 6092.3 | 6706.3 |
| 14 | CT18614 | 36437.7 | 29442.3 | 33767.0 | 113114.0 | 85550.7 | 69737.7 | 8519.3 | 6016.7 | 5727.3 |
| 15 | CT18615 | 38293.0 | 36513.7 | 27751.7 | 120592.3 | 105768.7 | 69824.7 | 8754.0 | 6246.0 | 8121.0 |
| 16 | CT19561 | 32430.3 | 28586.3 | 30350.3 | 118368.3 | 78387.7 | 63584.7 | 8206.3 | 5331.7 | 5347.3 |
| 17 | Pokalli | 38601.7 | 35939.0 | 34073.0 | 110153.7 | 101801.0 | 66450.0 | 7965.0 | 6195.0 | 5872.3 |
| 18 | CT6946 | 32775.0 | 39475.0 | 33934.7 | 113465.3 | 103078.7 | 80911.3 | 7020.3 | 7348.3 | 6919.0 |
| 19 | FED 2000 | 27681.0 | 30525.3 | 29778.0 | 98603.7 | 94302.3 | 89829.3 | 6512.7 | 5760.3 | 6686.3 |
| 20 | FEDE 21 | 39456.3 | 27110.3 | 27040.7 | 108174.7 | 84067.7 | 64988.0 | 8285.3 | 5524.0 | 5744.3 |
| 21 | FED 473 | 28827.0 | 38143.7 | 40754.7 | 100380.0 | 115572.0 | 123147.7 | 7653.7 | 8336.3 | 10244.0 |
| 22 | FED CARE | 35646.0 | 32942.7 | 30914.0 | 126946.7 | 114115.3 | 71038.7 | 8459.3 | 7196.7 | 5839.7 |
| 23 | HHZ 12 | 35964.3 | 35454.3 | 18419.7 | 93550.3 | 94252.0 | 41974.0 | 6117.3 | 6398.3 | 2577.7 |
| 24 | HHZ 1 | 39779.0 | 33769.7 | 38950.7 | 115196.0 | 97663.0 | 93206.3 | 8844.7 | 6887.3 | 8080.7 |
| 25 | IR04A115 | 32348.7 | 35719.7 | 35662.7 | 111653.3 | 117154.7 | 97795.7 | 7263.0 | 8619.0 | 10121.0 |
| 26 | IR05F102 | 31579.7 | 27937.3 | 40397.3 | 99725.7 | 80961.3 | 71270.0 | 6708.0 | 5224.3 | 6670.7 |
| 27 | IR05N412 | 36128.7 | 33679.7 | 34536.0 | 116079.7 | 100662.0 | 56359.0 | 9543.7 | 7739.0 | 5660.0 |
| 28 | IR06N155 | 28924.3 | 33241.3 | 11850.3 | 70331.7 | 70267.7 | 19455.7 | 6012.0 | 6430.3 | 1840.0 |
| 29 | IR07F102 | 36581.7 | 39490.0 | 34823.7 | 120920.7 | 111707.3 | 85656.3 | 8019.0 | 7567.0 | 6695.0 |
| 30 | IR07F287 | 31484.3 | 24032.3 | 23276.3 | 128855.3 | 76504.0 | 64106.7 | 9171.0 | 4587.0 | 4859.0 |
| 31 | IR07K142 | 30804.7 | 33132.3 | 24287.3 | 97072.3 | 108091.7 | 49185.7 | 6881.7 | 6533.0 | 4521.7 |
| 32 | IR08A172 | 38046.0 | 26678.3 | 28034.3 | 139451.7 | 91755.3 | 47874.0 | 9996.7 | 4925.7 | 4349.3 |
| 33 | IR08N136 | 23888.7 | 32248.3 | 30304.7 | 76254.7 | 89178.0 | 77145.7 | 5201.0 | 5680.3 | 5785.3 |
| 34 | IR09A130 | 28631.7 | 25843.3 | 28284.3 | 107565.3 | 78448.7 | 63963.0 | 7065.3 | 4350.0 | 4811.7 |
| 35 | IR09F436 | 23128.0 | 19850.3 | 25678.0 | 73478.7 | 42217.0 | 20360.3 | 5689.3 | 3124.0 | 1881.0 |
| 36 | IR09L179 | 22150.3 | 21962.7 | 13008.3 | 61139.0 | 58056.0 | 24675.3 | 5323.0 | 5234.0 | 2054.3 |
| 37 | IR09L324 | 32404.7 | 28970.0 | 23923.0 | 114769.7 | 100464.3 | 50038.7 | 7614.0 | 6513.3 | 3866.3 |
| 38 | IR09L337 | 28937.7 | 17875.0 | 25992.0 | 74243.3 | 42960.3 | 74356.7 | 5673.3 | 2396.0 | 6833.3 |
| 39 | IR09N537 | 36810.3 | 42692.3 | 23301.7 | 125572.7 | 112066.3 | 47395.0 | 9555.3 | 8511.3 | 3953.3 |
| 40 | IR10A134 | 20850.0 | 17565.0 | 25679.7 | 51729.0 | 42361.7 | 63448.3 | 3881.0 | 3217.3 | 5506.7 |
| 41 | IR10N230 | 32816.0 | 28833.0 | 33548.0 | 97325.7 | 72792.0 | 65065.7 | 6813.7 | 4543.3 | 4487.0 |
| 42 | IR49830 | 37851.7 | 28667.0 | 30108.3 | 110114.0 | 67737.0 | 74718.3 | 8359.7 | 4364.0 | 6288.0 |
| 43 | IR6-PAK | 28671.0 | 27130.7 | 20193.0 | 99572.3 | 82524.0 | 46321.0 | 7603.3 | 5140.3 | 3856.0 |
| 44 | IR64-NIL | 32667.7 | 38669.0 | 36857.3 | 128752.7 | 129059.7 | 104229.7 | 8560.3 | 9982.3 | 9523.7 |
| 45 | IR65482 | 33847.3 | 28934.0 | 24575.7 | 107205.7 | 73306.3 | 56947.0 | 6382.3 | 5011.7 | 4787.7 |
| 46 | IR65600 | 33434.3 | 28764.0 | 33320.3 | 108817.3 | 74708.3 | 67021.3 | 7513.0 | 4689.3 | 6185.0 |
| 47 | IR70213 | 32773.3 | 40320.7 | 33809.3 | 114867.0 | 105555.7 | 80666.0 | 7269.7 | 7858.7 | 7419.0 |
| 48 | IR74371 | 40845.7 | 39645.3 | 29359.3 | 114377.7 | 95927.0 | 72657.0 | 7239.3 | 6438.3 | 6092.7 |
| 49 | IR75483 | 34579.7 | 39723.3 | 28261.3 | 101673.7 | 88487.0 | 61184.3 | 7079.7 | 7229.7 | 6028.7 |
| 50 | IR78049 | 35752.7 | 32792.3 | 34307.3 | 130673.3 | 95906.0 | 71576.7 | 9434.0 | 6441.7 | 6929.3 |
| 51 | IR78221 | 32604.3 | 27360.3 | 30641.7 | 122979.3 | 70814.7 | 54528.7 | 9038.3 | 4122.3 | 4887.0 |
| 52 | IR78222 | 37762.0 | 33101.7 | 33201.0 | 117093.0 | 80291.7 | 67297.3 | 9162.0 | 6176.7 | 6762.3 |
| 53 | IR85411 | 32421.0 | 33474.7 | 39408.0 | 101524.7 | 109995.0 | 110434.7 | 6669.3 | 7153.0 | 10128.3 |
| 54 | IR85422 | 34053.0 | 33478.0 | 37567.3 | 131531.3 | 101744.7 | 113466.0 | 10693.0 | 6349.0 | 8641.7 |
| 55 | IR85427 | 39797.0 | 31181.0 | 29168.7 | 124492.3 | 77959.3 | 60825.3 | 9340.3 | 5560.7 | 6422.7 |
| 56 | IR86052 | 38229.7 | 27758.3 | 29319.7 | 128852.0 | 81271.3 | 74554.7 | 9160.3 | 5439.3 | 4928.3 |
| 57 | IR86126 | 37094.0 | 23590.7 | 15738.3 | 97833.0 | 59607.0 | 37570.0 | 7546.3 | 4242.0 | 2972.3 |
| 58 | IR86174 | 34795.3 | 38572.0 | 31857.7 | 122291.3 | 96476.0 | 109813.7 | 8810.3 | 6897.0 | 8565.7 |
| 59 | IR86174 | 34150.0 | 28839.7 | 37222.3 | 119965.3 | 78840.3 | 86204.0 | 9479.0 | 4704.0 | 7492.3 |
| 60 | IR86174 | 31980.3 | 28306.7 | 27242.0 | 105910.7 | 88192.0 | 59797.0 | 7905.3 | 5432.7 | 4712.0 |
| 61 | IR86635 | 41336.0 | 34482.0 | 44686.0 | 155452.3 | 102896.3 | 113996.3 | 11426.0 | 6967.3 | 9791.3 |
| 62 | IR88633 | 32420.7 | 30009.3 | 30285.3 | 106553.7 | 101115.7 | 72085.0 | 6923.3 | 6690.7 | 6291.3 |
| 63 | IR93323 | 32267.0 | 31323.7 | 29631.3 | 118448.3 | 91195.0 | 68156.3 | 8356.7 | 6634.0 | 4752.7 |
| 64 | Geumg | 38324.3 | 28702.0 | 40687.7 | 133385.7 | 81919.0 | 119839.7 | 10576.7 | 6233.7 | 11888.3 |
| 65 | IRRI 123 | 29565.7 | 29208.7 | 20172.0 | 74126.7 | 82618.0 | 39898.7 | 4863.0 | 5920.7 | 4200.0 |
| 66 | IRRI 152 | 28798.3 | 25523.0 | 26522.0 | 97314.7 | 70960.7 | 37399.0 | 7169.3 | 4687.3 | 3610.3 |
| 67 | IRRI 154 | 30661.7 | 32538.7 | 43397.3 | 89295.7 | 100801.3 | 109888.3 | 5815.7 | 6392.3 | 10300.7 |
| 68 | IRRI 157 | 32224.3 | 28141.0 | 33630.3 | 121135.3 | 95650.7 | 86218.3 | 7622.0 | 5848.7 | 6936.7 |
| 69 | MIL 240 | 36459.0 | 35963.3 | 44750.7 | 113176.7 | 103350.3 | 100861.3 | 7703.3 | 7063.0 | 10247.3 |
| 70 | MTU1010 | 34653.0 | 34865.7 | 31603.3 | 134771.0 | 99820.0 | 91502.0 | 8431.0 | 6297.7 | 6997.7 |
| 71 | PALMAR | 38301.3 | 37395.7 | 42895.7 | 111973.3 | 116699.0 | 117678.3 | 7671.0 | 8156.7 | 9765.7 |
| 72 | WAB | 31439.0 | 33280.3 | 37771.0 | 121332.7 | 89558.7 | 107915.3 | 8234.7 | 6275.0 | 8140.0 |
| 73 | Thad | 30590.3 | 26816.3 | 28996.7 | 91831.0 | 57767.0 | 51067.3 | 6190.0 | 4188.0 | 5323.0 |
| 74 | Rex | 26177.3 | 22624.0 | 21150.0 | 76144.0 | 49713.3 | 28355.7 | 5929.3 | 3296.3 | 2814.7 |
|  | **Means** | **33308.0** | **31261.1** | **30282.8** | **108884.8** | **89089.3** | **72119.2** | **7759.8** | **5998.9** | **6239.3** |

Table S5. Chlorophyll, flavonoids, anthocyanin, and nitrogen balance index of 74 rice genotypes under control (C), moderate salt stress (MSS) and high salt stress (HSS), measured 37 days after sowing. Each value represents the mean of four replications.

| Physiological Parameters | | | | | | | | | | | | | | |
| --- | --- | --- | --- | --- | --- | --- | --- | --- | --- | --- | --- | --- | --- | --- |
| S. No | Genotypes | Chlorophyll, µg cm^-2^ | | | Flavonoids | | | Anthocyanin | | | Nitrogen balance index | | | |
|  |  | C | MSS | HSS | C | MSS | HSS | C | MSS | HSS | C | MSS | HSS |  |
| 1 | 12DS-15 | 19.4 | 20.9 | 13.2 | 1.0 | 1.0 | 0.8 | 0.2 | 0.2 | 0.2 | 20.3 | 20.5 | 13.8 |  |
| 2 | 12DS-25 | 21.2 | 25.6 | 23.2 | 1.0 | 1.1 | 1.1 | 0.1 | 0.2 | 0.2 | 21.7 | 23.5 | 21.5 |  |
| 3 | 75-1-127 | 22.9 | 26.6 | 18.9 | 1.0 | 1.2 | 0.8 | 0.1 | 0.2 | 0.2 | 23.9 | 22.6 | 24.5 |  |
| 4 | Apo | 19.4 | 23.1 | 20.7 | 1.2 | 1.2 | 1.2 | 0.2 | 0.2 | 0.1 | 16.7 | 18.7 | 19.0 |  |
| 5 | BR47 | 27.7 | 30.2 | 27.5 | 1.0 | 1.1 | 1.2 | 0.1 | 0.1 | 0.1 | 26.2 | 26.9 | 22.4 |  |
| 6 | COL XXI | 19.5 | 27.8 | 22.4 | 1.1 | 1.1 | 0.9 | 0.2 | 0.1 | 0.1 | 18.2 | 24.0 | 24.7 |  |
| 7 | CT18233 | 23.5 | 26.2 | 29.1 | 1.1 | 1.2 | 1.3 | 0.2 | 0.1 | 0.1 | 21.9 | 21.6 | 23.8 |  |
| 8 | CT18237 | 27.2 | 26.5 | 22.5 | 1.2 | 1.1 | 1.0 | 0.1 | 0.1 | 0.2 | 23.2 | 24.7 | 21.8 |  |
| 9 | CT18244 | 15.0 | 19.0 | 20.6 | 1.1 | 1.1 | 1.1 | 0.2 | 0.2 | 0.2 | 14.0 | 17.0 | 16.7 |  |
| 10 | CT18245 | 15.0 | 29.7 | 29.3 | 1.0 | 1.1 | 1.3 | 0.2 | 0.1 | 0.2 | 14.8 | 26.9 | 24.0 |  |
| 11 | CT18247 | 21.9 | 26.3 | 24.8 | 1.0 | 1.0 | 1.1 | 0.2 | 0.1 | 0.2 | 21.8 | 25.6 | 23.4 |  |
| 12 | CT18372 | 21.0 | 21.2 | 12.6 | 1.0 | 1.1 | 0.9 | 0.2 | 0.2 | 0.2 | 19.9 | 18.8 | 14.0 |  |
| 13 | NB | 9.2 | 13.6 | 8.0 | 0.9 | 1.0 | 1.1 | 0.3 | 0.2 | 0.3 | 10.3 | 13.9 | 7.5 |  |
| 14 | CT18614 | 20.7 | 20.8 | 8.8 | 1.1 | 1.2 | 0.9 | 0.2 | 0.2 | 0.3 | 18.3 | 15.1 | 9.3 |  |
| 15 | CT18615 | 22.1 | 24.3 | 16.9 | 1.0 | 1.1 | 1.0 | 0.2 | 0.2 | 0.2 | 21.0 | 21.1 | 17.3 |  |
| 16 | CT19561 | 20.1 | 26.6 | 21.2 | 0.9 | 1.0 | 1.0 | 0.1 | 0.2 | 0.2 | 21.9 | 26.2 | 20.1 |  |
| 17 | Pokalli | 16.8 | 17.6 | 13.9 | 1.1 | 1.0 | 1.0 | 0.2 | 0.2 | 0.2 | 15.3 | 16.8 | 8.3 |  |
| 18 | CT6946 | 15.2 | 17.1 | 16.5 | 1.1 | 1.0 | 1.0 | 0.6 | 0.2 | 0.2 | 14.5 | 16.9 | 16.1 |  |
| 19 | FED 2000 | 23.4 | 28.7 | 22.6 | 1.0 | 1.1 | 1.1 | 0.1 | 0.1 | 0.2 | 20.0 | 27.3 | 22.6 |  |
| 20 | FEDE 21 | 15.6 | 24.0 | 17.9 | 1.1 | 1.1 | 1.1 | 0.2 | 0.2 | 0.2 | 13.8 | 21.4 | 16.1 |  |
| 21 | FED 473 | 16.0 | 24.1 | 18.0 | 1.0 | 1.0 | 0.9 | 0.2 | 0.1 | 0.2 | 15.3 | 23.1 | 15.7 |  |
| 22 | FED CARE | 19.2 | 21.1 | 20.6 | 1.1 | 1.1 | 1.1 | 0.2 | 0.2 | 0.2 | 18.2 | 18.7 | 20.0 |  |
| 23 | HHZ 12 | 17.9 | 20.3 | 11.7 | 1.1 | 1.0 | 1.0 | 0.2 | 0.2 | 0.2 | 15.9 | 20.8 | 11.2 |  |
| 24 | HHZ 1 | 12.1 | 19.1 | 16.6 | 0.9 | 1.1 | 1.1 | 0.2 | 0.2 | 0.2 | 13.2 | 17.0 | 14.3 |  |
| 25 | IR04A115 | 23.1 | 27.8 | 17.5 | 1.2 | 1.2 | 1.0 | 0.2 | 0.1 | 0.1 | 19.9 | 26.1 | 16.7 |  |
| 26 | IR05F102 | 16.9 | 21.9 | 13.2 | 1.0 | 0.9 | 0.9 | 0.2 | 0.1 | 0.2 | 17.6 | 23.2 | 14.8 |  |
| 27 | IR05N412 | 17.7 | 19.8 | 15.3 | 1.1 | 1.0 | 1.0 | 0.2 | 0.2 | 0.2 | 17.2 | 19.7 | 15.8 |  |
| 28 | IR06N155 | 22.9 | 25.4 | 16.9 | 1.0 | 1.0 | 1.0 | 0.1 | 0.1 | 0.2 | 23.4 | 25.4 | 15.4 |  |
| 29 | IR07F102 | 17.0 | 22.0 | 18.9 | 1.0 | 1.0 | 1.1 | 0.2 | 0.2 | 0.2 | 17.5 | 21.7 | 15.0 |  |
| 30 | IR07F287 | 21.7 | 23.9 | 22.0 | 1.0 | 1.0 | 1.1 | 0.1 | 0.1 | 0.2 | 20.7 | 23.3 | 17.3 |  |
| 31 | IR07K142 | 26.1 | 33.3 | 21.9 | 1.1 | 1.1 | 1.0 | 0.1 | 0.1 | 0.2 | 23.6 | 30.3 | 22.2 |  |
| 32 | IR08A172 | 25.2 | 29.3 | 20.6 | 1.1 | 1.2 | 1.0 | 0.1 | 0.1 | 0.2 | 22.9 | 24.5 | 20.7 |  |
| 33 | IR08N136 | 20.2 | 26.4 | 22.1 | 1.1 | 1.1 | 1.1 | 0.2 | 0.1 | 0.2 | 18.9 | 23.6 | 19.9 |  |
| 34 | IR09A130 | 21.0 | 26.9 | 20.1 | 1.1 | 1.1 | 1.0 | 0.2 | 0.1 | 0.2 | 18.9 | 24.0 | 18.4 |  |
| 35 | IR09F436 | 23.6 | 29.8 | 14.4 | 1.0 | 0.9 | 1.0 | 0.1 | 0.1 | 0.2 | 23.2 | 34.1 | 14.2 |  |
| 36 | IR09L179 | 25.8 | 24.3 | 14.2 | 1.1 | 1.1 | 1.1 | 0.1 | 0.2 | 0.2 | 24.3 | 21.9 | 13.2 |  |
| 37 | IR09L324 | 23.4 | 29.3 | 21.4 | 1.1 | 1.2 | 1.0 | 0.2 | 0.1 | 0.2 | 22.0 | 24.8 | 15.4 |  |
| 38 | IR09L337 | 24.0 | 26.8 | 23.4 | 1.0 | 1.2 | 1.3 | 0.1 | 0.1 | 0.3 | 23.5 | 23.0 | 20.7 |  |
| 39 | IR09N537 | 21.0 | 24.8 | 14.5 | 1.1 | 1.1 | 1.0 | 0.2 | 0.1 | 0.2 | 20.1 | 23.7 | 13.9 |  |
| 40 | IR10A134 | 29.6 | 29.3 | 14.1 | 1.0 | 1.1 | 0.9 | 0.1 | 0.1 | 0.2 | 29.4 | 27.8 | 14.9 |  |
| 41 | IR10N230 | 24.0 | 25.9 | 18.6 | 1.1 | 1.1 | 1.1 | 0.1 | 0.2 | 0.1 | 21.6 | 23.8 | 16.9 |  |
| 42 | IR49830 | 24.4 | 29.2 | 24.7 | 1.2 | 1.1 | 1.1 | 0.2 | 0.1 | 0.2 | 19.9 | 27.9 | 22.2 |  |
| 43 | IR6-PAK | 20.6 | 26.2 | 21.6 | 1.1 | 1.1 | 1.1 | 0.2 | 0.1 | 0.2 | 18.7 | 25.4 | 19.8 |  |
| 44 | IR64-NIL | 17.8 | 21.2 | 20.8 | 1.1 | 1.1 | 1.1 | 0.2 | 0.2 | 0.2 | 18.3 | 18.7 | 18.1 |  |
| 45 | IR65482 | 27.6 | 28.4 | 20.9 | 1.1 | 1.0 | 1.0 | 0.1 | 0.1 | 0.2 | 26.7 | 28.4 | 21.1 |  |
| 46 | IR65600 | 26.5 | 23.8 | 26.0 | 1.2 | 1.0 | 1.3 | 0.1 | 0.2 | 0.3 | 22.7 | 23.1 | 17.7 |  |
| 47 | IR70213 | 26.8 | 27.1 | 25.6 | 1.0 | 1.3 | 1.1 | 0.1 | 0.1 | 0.2 | 25.7 | 20.7 | 22.7 |  |
| 48 | IR74371 | 27.0 | 26.9 | 23.0 | 1.0 | 1.1 | 1.1 | 0.1 | 0.2 | 0.1 | 26.8 | 23.2 | 21.2 |  |
| 49 | IR75483 | 25.9 | 22.3 | 16.1 | 1.1 | 1.0 | 0.9 | 0.1 | 0.2 | 0.2 | 24.8 | 23.1 | 17.9 |  |
| 50 | IR78049 | 23.5 | 21.6 | 17.8 | 1.2 | 1.0 | 1.0 | 0.2 | 0.2 | 0.1 | 20.6 | 20.9 | 17.7 |  |
| 51 | IR78221 | 30.5 | 31.6 | 20.4 | 1.2 | 1.1 | 0.9 | 0.1 | 0.1 | 0.2 | 25.3 | 28.4 | 21.0 |  |
| 52 | IR78222 | 26.4 | 29.2 | 21.6 | 1.1 | 1.0 | 0.9 | 0.2 | 0.1 | 0.2 | 23.6 | 27.8 | 24.3 |  |
| 53 | IR85411 | 16.6 | 20.4 | 18.8 | 0.9 | 1.1 | 1.0 | 0.2 | 0.2 | 0.2 | 18.8 | 19.0 | 18.1 |  |
| 54 | IR85422 | 18.2 | 18.2 | 19.1 | 1.1 | 1.1 | 1.0 | 0.2 | 0.2 | 0.2 | 17.1 | 16.5 | 18.5 |  |
| 55 | IR85427 | 14.0 | 13.6 | 7.3 | 1.2 | 1.1 | 1.0 | 0.2 | 0.2 | 0.3 | 11.3 | 12.3 | 7.1 |  |
| 56 | IR86052 | 28.0 | 28.1 | 23.3 | 1.1 | 1.1 | 1.0 | 0.1 | 0.1 | 0.1 | 25.2 | 26.2 | 22.7 |  |
| 57 | IR86126 | 26.0 | 28.6 | 18.3 | 1.0 | 1.1 | 1.0 | 0.1 | 0.1 | 0.2 | 25.1 | 25.5 | 17.0 |  |
| 58 | IR86174 | 24.4 | 26.8 | 31.1 | 1.1 | 1.1 | 1.1 | 0.2 | 0.1 | 0.1 | 22.4 | 23.2 | 29.1 |  |
| 59 | IR86174 | 14.8 | 19.2 | 21.6 | 1.1 | 1.2 | 1.1 | 0.2 | 0.2 | 0.2 | 13.1 | 16.0 | 19.5 |  |
| 60 | IR86174 | 17.5 | 19.7 | 20.4 | 1.0 | 1.1 | 1.2 | 0.2 | 0.2 | 0.2 | 17.4 | 18.8 | 16.9 |  |
| 61 | IR86635 | 20.7 | 20.9 | 26.0 | 1.0 | 1.1 | 1.2 | 0.2 | 0.2 | 0.1 | 21.3 | 17.7 | 21.3 |  |
| 62 | IR88633 | 17.5 | 23.0 | 18.3 | 0.9 | 1.2 | 1.0 | 0.2 | 0.2 | 0.2 | 18.4 | 19.6 | 17.1 |  |
| 63 | IR93323 | 20.3 | 24.2 | 16.7 | 1.1 | 1.1 | 1.0 | 0.2 | 0.2 | 0.2 | 18.8 | 23.1 | 16.9 |  |
| 64 | Geumg | 21.7 | 28.3 | 17.9 | 0.9 | 1.1 | 1.0 | 0.1 | 0.1 | 0.2 | 24.0 | 26.6 | 17.1 |  |
| 65 | IRRI 123 | 20.1 | 25.6 | 19.0 | 1.1 | 1.2 | 1.1 | 0.1 | 0.1 | 0.2 | 20.2 | 21.8 | 17.3 |  |
| 66 | IRRI 152 | 19.3 | 20.0 | 19.0 | 1.1 | 1.2 | 1.0 | 0.2 | 0.2 | 0.2 | 18.0 | 16.2 | 19.5 |  |
| 67 | IRRI 154 | 17.8 | 35.8 | 23.8 | 1.1 | 1.5 | 1.1 | 0.2 | 0.1 | 0.2 | 15.5 | 25.3 | 20.6 |  |
| 68 | IRRI 157 | 22.9 | 28.7 | 19.5 | 1.0 | 1.1 | 1.1 | 0.1 | 0.1 | 0.2 | 23.0 | 27.1 | 16.3 |  |
| 69 | MIL 240 | 18.1 | 26.3 | 13.0 | 1.0 | 1.2 | 1.1 | 0.2 | 0.2 | 0.2 | 18.5 | 21.2 | 12.2 |  |
| 70 | MTU1010 | 26.8 | 27.7 | 24.9 | 1.3 | 1.1 | 1.2 | 0.1 | 0.1 | 0.2 | 21.0 | 25.3 | 23.4 |  |
| 71 | PALMAR | 19.8 | 27.6 | 20.4 | 1.2 | 1.3 | 1.0 | 0.2 | 0.2 | 0.2 | 17.0 | 21.8 | 20.0 |  |
| 72 | WAB | 22.8 | 25.2 | 24.7 | 1.0 | 1.0 | 1.1 | 0.1 | 0.1 | 0.1 | 22.1 | 25.4 | 22.3 |  |
| 73 | Thad | 32.7 | 33.1 | 26.3 | 1.2 | 1.2 | 1.0 | 0.1 | 0.1 | 0.2 | 27.8 | 28.7 | 26.1 |  |
| 74 | Rex | 22.0 | 26.2 | 20.1 | 1.1 | 1.0 | 1.0 | 0.2 | 0.2 | 0.2 | 20.2 | 25.9 | 20.8 |  |
|  | **Means** | **21.4** | **24.9** | **19.7** | **1.1** | **1.1** | **1.0** | **0.2** | **0.2** | **0.2** | **20.2** | **22.7** | **18.3** |  |
